# Homelessness, unstable housing, and risk of HIV and hepatitis C virus acquisition among people who inject drugs: a systematic review and meta-analysis

**DOI:** 10.1016/S2468-2667(21)00013-X

**Published:** 2021-03-26

**Authors:** Chiedozie Arum, Hannah Fraser, Andreea Adelina Artenie, Sandra Bivegete, Adam Trickey, Michel Alary, Jacquie Astemborski, Jennifer Iversen, Aaron G Lim, Louis MacGregor, Meghan Morris, Jason J Ong, Lucy Platt, Rachel Sack-Davis, Daniela K van Santen, Sunil S Solomon, Vana Sypsa, Jorge Valencia, Wijnand Van Den Boom, Josephine G Walker, Zoe Ward, Jack Stone, Peter Vickerman, Homelessness, HIV, and HCV Review Collaborative Group, Peter Cherutich, Kora Debeck, Paul Dietze, Kostyantyn Dumchev, Kanna Hayashi, Margaret Hellard, Matthew Hickman, Vivian Hope, Ali Judd, Martin Kåberg, Ann E. Kurth, Pascale Leclerc, Lisa Maher, Shruti H. Mehta, Kimberly A Page, Maria Prins, Catherine S. Todd, Steffanie A. Strathdee

**Affiliations:** a Population Health Sciences, University of Bristol, Bristol, UK; b Centre de recherche du CHU de Québec, Université Laval, Quebec City, QC, Canada; c Département de médecine sociale et préventive, Université Laval, Quebec City, QC, Canada; d Institut national de santé publique du Québec, Québec, QC, Canada; e Department of Epidemiology, Johns Hopkins Bloomberg School of Public Health, Baltimore, MD, USA; f Kirby Institute for Infection and Immunity, UNSW Sydney, NSW, Australia; g Department of Epidemiology and Biostatistics, University of California San Francisco, San Francisco, CA, USA; h Department of Clinical Research, London School of Hygiene & Tropical Medicine, London, UK; i Faculty of Public Health and Policy, London School of Hygiene & Tropical Medicine, London, UK; j Burnet Institute, Melbourne, VIC, Australia; k Department of Infectious Disease Research and Prevention, Public Health Service of Amsterdam, Amsterdam, Netherlands; l Department of Epidemiology and Preventive Medicine, Monash University, Melbourne, VIC, Australia; m Department of Hygiene, Epidemiology and Medical Statistics, Medical School, National and Kapodistrian University of Athens, Athens, Greece; n Harm Reduction Unit “SMASD”, Department of Addictions and Mental Health, Madrid, Spain

## Abstract

**Background:**

People who inject drugs (PWID) are at increased risk for HIV and hepatitis C virus (HCV) infection and also have high levels of homelessness and unstable housing. We assessed whether homelessness or unstable housing is associated with an increased risk of HIV or HCV acquisition among PWID compared with PWID who are not homeless or are stably housed.

**Methods:**

In this systematic review and meta-analysis, we updated an existing database of HIV and HCV incidence studies published between Jan 1, 2000, and June 13, 2017. Using the same strategy as for this existing database, we searched MEDLINE, Embase, and PsycINFO for studies, including conference abstracts, published between June 13, 2017, and Sept 14, 2020, that estimated HIV or HCV incidence, or both, among community-recruited PWID. We only included studies reporting original results without restrictions to study design or language. We contacted authors of studies that reported HIV or HCV incidence, or both, but did not report on an association with homelessness or unstable housing, to request crude data and, where possible, adjusted effect estimates. We extracted effect estimates and pooled data using random-effects meta-analyses to quantify the associations between recent (current or within the past year) homelessness or unstable housing compared with not recent homelessness or unstable housing, and risk of HIV or HCV acquisition. We assessed risk of bias using the Newcastle-Ottawa Scale and between-study heterogeneity using the I^2^ statistic and p value for heterogeneity.

**Findings:**

We identified 14 351 references in our database search, of which 392 were subjected to full-text review alongside 277 studies from our existing database. Of these studies, 55 studies met inclusion criteria. We contacted the authors of 227 studies that reported HIV or HCV incidence in PWID but did not report association with the exposure of interest and obtained 48 unpublished estimates from 21 studies. After removal of duplicate data, we included 37 studies with 70 estimates (26 for HIV; 44 for HCV). Studies originated from 16 countries including in North America, Europe, Australia, east Africa, and Asia. Pooling unadjusted estimates, recent homelessness or unstable housing was associated with an increased risk of acquiring HIV (crude relative risk [cRR] 1·55 [95% CI 1·23–1·95; p=0·0002]; I^2^= 62·7%; n=17) and HCV (1·65 [1·44–1·90; p<0·0001]; I^2^= 44·8%; n=28]) among PWID compared with those who were not homeless or were stably housed. Associations for both HIV and HCV persisted when pooling adjusted estimates (adjusted relative risk for HIV: 1·39 [95% CI 1·06–1·84; p=0·019]; I^2^= 65·5%; n=9; and for HCV: 1·64 [1·43–1·89; p<0·0001]; I^2^= 9·6%; n=14). For risk of HIV acquisition, the association for unstable housing (cRR 1·82 [1·13–2·95; p=0·014]; n=5) was higher than for homelessness (1·44 [1·13–1·83; p=0·0036]; n=12), whereas no difference was seen between these outcomes for risk of HCV acquisition (1·72 [1·48–1·99; p<0·0001] for unstable housing, 1·66 [1·37–2·00; p<0·0001] for homelessness).

**Interpretation:**

Homelessness and unstable housing are associated with increased risk of HIV and HCV acquisition among PWID. Our findings support the development of interventions that simultaneously address homelessness and unstable housing and HIV and HCV transmission in this population.

**Funding:**

National Institute for Health Research, National Institute on Drug Abuse, National Institute of Allergy and Infectious Diseases, and Commonwealth Scholarship Commission.

## Introduction

Globally, HIV and viral hepatitis are leading causes of mortality,^1^, ^2^ with people who inject drugs (PWID) being highly susceptible to HIV and hepatitis C virus (HCV) infection.^3^, ^4^, ^5^, ^6^ Over 2018–30, an estimated 43% of global HCV transmission is projected to be attributed to unsafe injecting practices among PWID.^6^ Approximately 8% of new HIV infections globally and 20% outside sub-Saharan Africa occur among PWID.^5^ Although effective prevention and treatment interventions exist for reducing the transmission of HIV and HCV among PWID,^7^, ^8^, ^9^, ^10^, ^11^ coverage remains low globally.^12^ Additionally, PWID are usually exposed to multiple adverse environments (eg, incarceration and homelessness) that can increase their risk of HIV and HCV infection.^13^, ^14^, ^15^, ^16^ Homelessness, defined by the Institute of Global Homelessness as lacking access to adequate housing, and unstable housing, typically defined as being without fixed housing, are widely acknowledged as important risk factors for acquisition of HCV or HIV infection,^13^, ^14^, ^15^, ^16^ and have been implicated in a series of HIV outbreaks among PWID over the past 10 years.^17^

**Research in context**

**Evidence before this study**

We searched PubMed, with no language restrictions, for publications since database inception up to July 9, 2020, using the terms (“HIV” OR “hepatitis C” OR “HCV”) AND (“homelessness” OR “unstable housing”) AND (“inject drugs” OR “injecting drug” OR “substance abuse” OR “intravenous/epidemiology [MeSH]” OR “substance-related disorders/epidemiology [MeSH]”). We identified studies that suggested that recent homelessness or unstable housing was negatively associated with initiation and adherence to opioid substitution treatment (OST; eight studies), use of needle-syringe programmes (two studies), initiation and adherence to HIV and hepatitis C virus (HCV) treatment and their outcomes (20 studies), cessation of injecting drug use (three studies), and access to primary care (one study). Conversely, recent homelessness or unstable housing was positively associated with prevalent HIV and HCV infection (18 studies), stimulant injecting (ten studies), increased injecting frequency (six studies), increased duration of injecting (one study), exposure to physical and sexual violence (nine studies), use of emergency services and admission to hospital (six studies), overdose (three studies), and mortality (three studies). Our search also identified studies that reported a positive association between homelessness or unstable housing and unemployment (two studies), food insecurity (one study), incarceration (three studies), high-risk sexual behaviours such as commercial sex (15 studies), and high-risk injecting practices such as receptive syringe sharing and public injecting (27 studies). We identified a systematic review on factors associated with injecting-related risk behaviours among people who inject drugs (PWID) that implicated homelessness and unstable housing as important determinants of risky injecting practices, such as increased injecting frequency, public injecting, syringe sharing, and stimulant injecting. Additionally, we found a systematic review of factors associated with HIV treatment adherence among PWID, which identified homelessness or unstable housing as a substantial structural barrier to adherence. We also found several studies linking homelessness or unstable housing with incident HIV and HCV infection or reinfection among PWID (21 studies). We also identified a study in which PWID who experienced increased housing stability over time had lower injecting frequency than those with decreasing housing stability. Furthermore, we identified a systematic review of outcomes associated with participation in Housing First programmes, which suggests that housing interventions are associated with reduced high-risk sexual behaviours and improved treatment outcomes among homeless populations with HIV.

**Added value of this study**

To our knowledge, this is the first systematic review and meta-analysis on the effect of recent homelessness or unstable housing on risk of HIV and HCV acquisition among PWID compared with PWID who are not homeless or who are stably housed. Our study also builds on previously published evidence through collating and pooling unpublished estimates, obtained by contacting authors of all identified studies of PWID that reported a measure of HIV or HCV incidence but not on our outcomes of interest. This resulted in an additional 21 studies being included in our review, nearly tripling the overall number of estimates included. We found that recent homelessness or unstable housing (current, or past 1–12 months) was associated with an increased risk of HIV and HCV acquisition and these associations mostly persisted in sensitivity analyses.

**Implications of all the available evidence**

Our review supports increasing evidence on the deleterious effect of housing instability on the health and social outcomes of PWID, specifically increased risk of HIV and HCV acquisition. Homeless and unstably housed PWID often face profound disadvantages and have multiple competing priorities. Therefore, a comprehensive approach that not only provides housing but also addresses many of the interlinked health and social concerns of this population is necessary to reduce HIV and HCV risk. Further research is needed to better understand how homelessness and unstable housing increases the risk of HIV and HCV acquisition, and what interventions could most effectively reduce this risk. This research would guide policies to help PWID attain and maintain housing stability.

Homelessness or unstable housing is a well established determinant of poor health outcomes and excess mortality,^18^, ^19^, ^20^, ^21^ affecting many PWID. Globally, an estimated 22% of PWID report experiencing homelessness or unstable housing in the past year.^4^ Relative to PWID living in stable housing, those who are homeless or unstably housed are more likely to engage in high-risk behaviours associated with HIV and HCV transmission, such as sex work, public injecting, and sharing of injection equipment.^22^, ^23^, ^24^ They also experience barriers to accessing drug addiction treatment^25^, ^26^ and HIV and HCV prevention and care.^23^, ^27^, ^28^ Studies exploring the perceptions of PWID around high-risk behaviours for HCV infection have further highlighted the detrimental effect of homelessness.^29^

Although multiple studies have reported associations between homelessness or unstable housing and incident HIV and HCV infection among PWID,^30^, ^31^, ^32^, ^33^, ^34^ to date, no systematic review has synthesised these data. We did a systematic review and meta-analysis to quantify the associations between homelessness or unstable housing and the risk of HIV and HCV acquisition among PWID.

## Methods

### Search strategy and selection criteria

In this systematic review and meta-analysis, we updated an existing database of HIV and HCV incidence studies published between Jan 1, 2000, and June 13, 2017. This reference database was compiled during a previous systematic review and meta-analysis.^35^ Using the same strategy developed previously,^35^ CA did a systematic literature search of MEDLINE, Embase, and PsycINFO for studies published between June 13, 2017, and Sept 14, 2020, including conference abstracts, without language restrictions. We used terms related to HIV infection, HCV infection, injecting drug use, and study designs that could be used to estimate incidence of HIV or HCV (a full list of search terms is in the appendix [pp 7–8]). We restricted our analysis to studies done among community-recruited (ie, not recruited in prisons) PWID (those with history of recent or ever injecting drug use) that assessed HIV or HCV incidence. We included studies if they had already assessed or were able to assess whether or not an association existed between recent homelessness or unstable housing and HIV or HCV incidence compared with PWID who were not recently homeless or were stably housed. We allowed for study-level differences in the definition of homelessness and unstable housing, and the timeframe definition of recent, which varied from currently to the past 1–12 months. We included studies that measured HIV or HCV incidence through longitudinal follow-up and testing or biological markers of recent infection (eg, anti-HCV avidity and BED assays for HCV and HIV).^33^, ^36^ We only included studies reporting original results without restrictions to design or language. We contacted authors of studies (including those published as conference abstracts) that reported estimates of HIV and HCV incidence, but did not report on the association with homelessness or unstable housing, to request data. We also contacted the lead investigators of other ongoing HIV and HCV incidence cohorts to request data. We requested crude and, where possible, adjusted effect estimates (preferably in the form of hazard ratios [HRs] and adjusting for opioid substitution therapy [OST] exposure, recent incarceration, and stimulant injecting), as done previously.^7^, ^8^, ^35^ Throughout this Article, we use the term unpublished estimates to refer to estimates that we calculated for this study from raw data obtained by contacting authors. Although these estimates of associations between homelessness or unstable housing and HIV or HCV acquisition had not been previously presented, the vast majority were calculated using raw data from studies with published estimates of HIV or HCV incidence. For these unpublished estimates, we cite the most recently published article that was based on the same cohort. Some of the cohorts included in this systematic review and meta-analysis extend over long periods of time. Estimates derived from the same cohort but based on different durations of follow-up were identified on the basis of matching study names, settings, or authors, or a combination of these. To avoid publication bias, we included only one estimate, retaining the one with the most person-years of follow-up overall.

We created an Endnote library (version X9) to catalogue the search results and to de-duplicate references. After removal of duplicates, CA screened the titles and abstracts of the studies to identify papers or reports that might contain relevant information. After finding discrepancies during an initial double-screening of a random 10% of studies, HF, JS, SB, AT, JGW, AGL, LM, JJO, and ZW double-screened all the titles and abstracts, with disagreements resolved by group discussion. CA screened the full texts of potentially relevant records to identify those that met the inclusion criteria. HF, JS, SB, AT, JGW, AGL, LM, JJO, and ZW double-screened these records with discrepancies resolved by group discussion. We used Google Translate to read non-English language papers.

This study is reported in accordance with PRISMA guidelines^37^ (appendix pp 1–2). Details of the methods were prespecified and documented in a protocol (appendix pp 3–6). No deviations from the protocol occurred.

### Data analysis

We sought unadjusted and adjusted summary estimates for our main analysis, as well as summaries of participant-level and study-level characteristics for our subgroup analyses, meta-regression, and sensitivity analyses. CA extracted data from selected studies into Microsoft Excel using a pre-defined data extraction spreadsheet (appendix p 8). JS, HF, and AAA double-checked the extracted data, with discrepancies resolved by group discussion. We extracted one unadjusted and adjusted effect measure from published papers, prioritising HRs, incidence rate ratios, and relative risks (RRs) over odds ratios (ORs). When an effect estimate was not reported, we calculated one from the available data. Consistent with previously published methods,^38^ we transformed ORs and their 95% CIs into RRs when incidence was high (>10 per 100 person-years).

We assessed the risk of bias for each estimate of HIV or HCV incidence for each study using the Newcastle-Ottawa Scale.^39^ The scale allocates a maximum of 9 points indicating a low risk of bias depending on the selection of study participants, comparability of participants, and ascertainment of the outcome. To assess the comparability of participants, we examined whether the reported effect estimates were adjusted for three variables that we identified a priori as being potentially confounding factors: OST exposure, history of incarceration, and history of stimulant injecting. This decision was based on studies reporting a lower risk of HIV and HCV infection among PWID on OST,^7^, ^8^ and an inverse association between homelessness or unstable housing and OST exposure.^30^, ^40^ Conversely, some studies have indicated that PWID who have been recently incarcerated or who inject stimulants have a greater risk of HIV and HCV infection^41^, ^42^ and are more likely to be homeless or unstably housed.^23^, ^43^, ^44^ We gave 1 point to studies that adjusted for OST exposure and 1 point to those that adjusted for history of incarceration or stimulant injecting, or both. To assess the risk of bias in unpublished estimates, we consulted the corresponding published paper or papers. For all records, risk of bias was independently assessed by two authors (CA and JS or HF) with discrepancies resolved by discussion with AAA.

We combined study-specific effect estimates using random-effects meta-analysis, because we anticipated high between-study variation. We log-transformed effect measures and their SEs, with crude and adjusted estimates pooled separately. We plotted the unadjusted estimates onto a forest plot and assessed heterogeneity by inspection of these plots, and using the *I*^2^ statistic and p value for heterogeneity.^45^

We did separate sensitivity analyses to assess the robustness of pooled estimates by restricting the meta-analysis to: studies that reported HRs (most common effect measure), studies at low-to-moderate risk of bias (Newcastle-Ottawa Scale score of ≥6), longitudinal studies, adjusted estimates, and studies in which 90% or more of participants had injected recently. The confounding factors included in the adjusted analyses varied between studies. We used funnel plot and Egger's test for funnel plot asymmetry to assess the risk of bias due to missing results for each outcome.

We did subgroup and random-effects meta-regression analyses to investigate potential sources of heterogeneity, as prespecified in the protocol (appendix p 5). These potential sources of heterogeneity included the baseline characteristics of participants (proportion female, proportion ever incarcerated, proportion recently incarcerated, proportion prescribed OST, mean or median age, time since starting injecting drug use, and HIV or HCV prevalence), study characteristics (publication status, study design, years of study, study duration, effect measure, timeframe for defining recent homelessness or unstable housing), and geographical and economic region.

We did all analyses separately for HIV and HCV. We did all data analysis using Stata (version 15.1).

### Role of the funding source

The funders of the study had no role in the study design, data collection, data analysis, data interpretation, or writing of the report.

## Results

**Figure 1:**
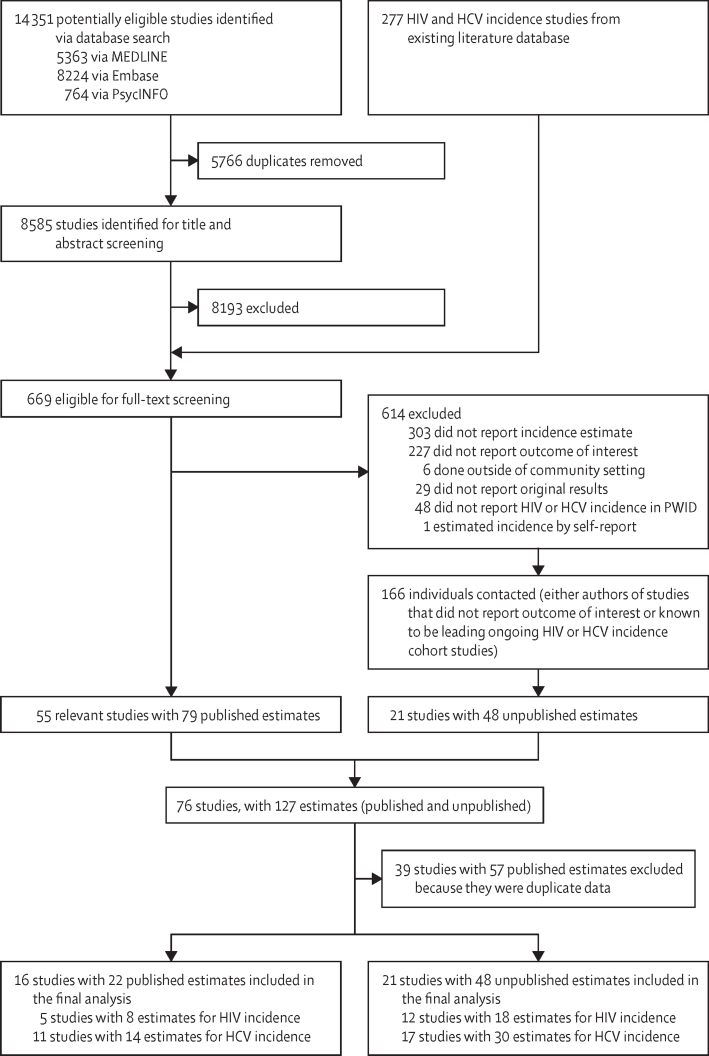
Study selection Unpublished estimates are those that have been calculated from raw data for this study and have not been presented in previously published studies. HCV=hepatitis C virus. PWID=people who inject drugs.

Through our database searches of HIV and HCV incidence studies published between June 13, 2017, and Sept 14, 2020, we identified 14 351 potentially eligible studies, of which 5766 were duplicates (figure 1). Initial screening of titles and abstracts of the remaining 8585 studies resulted in 392 studies eligible for full-text review. We retrieved 277 additional studies from our existing database of HIV and HCV incidence studies (search from Jan 1, 2000, to June 13, 2017),^35^ which resulted in 669 references for full-text review. Of these, 55 studies (79 estimates) met the inclusion criteria. Additionally, we identified 227 studies that measured HIV or HCV incidence among PWID but did not report an association with the exposure of interest. We contacted the authors of these studies and known lead investigators of other ongoing HIV and HCV incidence cohorts to request data. 48 unpublished estimates (from 21 studies) were obtained from these sources; 43 were from published studies reporting HIV and HCV incidences, but not reporting the exposure of interest, four were updates of previously published estimates, and one was from an ongoing study that had not yet been published. Overall, we identified 76 studies with 127 estimates that met the inclusion criteria for our review. Of these, we excluded 39 studies with 57 estimates as duplicate data (details of excluded studies are shown in the appendix [pp 9–10]).

The characteristics of studies included in the final analysis are shown in table 1, which includes 37 studies, of which eight reported data for both HIV and HCV incidence, originating from 16 countries, giving 70 effect estimates (22 published and 48 unpublished estimates) done between 1986 and 2020. There were 29 314 participants across HIV studies and 21 842 participants across HCV studies. There were 32 longitudinal studies (15 for HIV; 25 for HCV) and five cross-sectional studies (two for HIV; three for HCV); total person-years of follow-up for HIV longitudinal studies was 51 977 person-years (data missing for three studies) and for HCV longitudinal studies was 22 370 person-years (data missing for four studies). Of included effect estimates, 49 measured the effect of recent homelessness (19 for HIV; 30 for HCV), while 21 measured the effect of recent unstable housing (seven for HIV; 14 for HCV), with the timeframe for recent defined as currently for 12 studies, 6 months or less for 19 studies, and less than 12 months for six studies. Although not all included studies provided an explicit definition for homelessness or unstable housing, homelessness was generally defined as living on the streets, in cars, and in abandoned houses,^41^, ^67^, ^69^, ^75^ whereas unstable housing was defined as being without fixed housing,^75^ including so-called sofa-surfing and living on the street, in hostels, in guesthouses, in shelters, in single-room occupancies, or in short-term rentals (typically motels, hotels, rooming or multitenant houses, or rented rooms).^47^, ^48^, ^68^, ^75^ These definitions were not mutually exclusive, such that unstable housing often incorporated homelessness. The overall proportion of female participants was 4127 (15·6%) of 26 384 participants for HIV studies (data missing for two studies) and 5332 (28·6%) of 18 628 for HCV studies (data missing for five studies). We saw large differences in baseline prevalence of HIV (0·0–43·6%) and HCV (11·5–85·0%). In total, there were 1224 incident HIV infections (data missing for two studies), with HIV incidence ranging from 0·9–20·0 per 100 person-years, and 1051 incident HCV infections (data missing for three studies), with HCV incidence ranging from 0·9–64·5 per 100 person-years. Further details on study characteristics are in the appendix (pp 20–28).

**Table 1:** Study characteristics of 37 studies, of which eight report data for HIV and HCV incidence, included in systematic review and meta-analysis

	**Study period**	**Location (city, country)**	**Study design**	**Effect measured and definition**	**HCV or HIV estimate**	**Sample size**	**Incidence (per 100 person-years)**	**Effect estimates**	**Confounding factors included in adjusted estimates**	**Newcastle-Ottawa Scale score**
Alary (unpublished estimate)	2003–18	Province of Quebec and Ottawa, Canada	Retrospective cohort^46^	Unstable housing in the past 6 months	HIV and HCV	HIV: 1930 HCV: 814	HIV: 1·08 HCV: 20·2	HIV: HR 1·26 (0·83–1·93);aHR 0·98 (0·62–1·53) HCV: HR 1·64 (1·34–2·00);aHR 1·44 (1·16–1·78)	Living in jail within the past 6 months, OST exposure, using syringes used by someone else, cocaine being most often injected drug, injecting drugs every day, age ≥25 years, male gender, prostitution, urban sites	HIV: 7 HCV: 7
Artenie et al (2019)^47^	2004–17	Montreal, QC, Canada	Prospective cohort	Unstable housing in the past month	HCV	513	11·8	HR 2·34 (1·72–3·17);aHR 2·14 (1·54–2·96)	OAT dose and perceived adequacy, sex, duration of injection drug use, cocaine injection in the past month, incarceration in the past 3 or 6 months, previous HCV infection	8
Bruneau et al (2011)^48^	1992–2008	Montreal, QC, Canada	Prospective cohort	Unstable housing in the past 6 months	HIV	2137	3·3	HR 3·08 (2·22–4·28);aHR 2·07 (1·47–2·90)	Age ≥30 years, gender, cocaine use in the past month, heroin use in the past month, sharing syringes with a person known to be HIV positive, “booting”, having sex with a person known to be HIV positive, period of recruitment, NEP participation, obtaining 100% of syringes from a safe source	7
Craine et al (2009)^49^	2004–06	Newport and Calidicot, Cardiff and Barry, Bridgend, Neath & Porth Talbot, Swansea, Merthyr Tydfil, Pontypridd, Rhydfelin, Treorchy, Aberdare, Abergavenny, UK	Prospective cohort	Homelessness in the past 12 months	HCV	286	5·9	IRR 4·41 (1·60–12·5);aIRR 2·9 (1·02–8·28)	In OST at follow-up, any equipment sharing in the past year, sharing needles and syringes in past year, population size of region	7
Debeck (unpublished estimate)	2005–16	Vancouver, BC, Canada	Prospective cohort^50^	Homelessness in the past 6 months	HIV and HCV	HIV: 476 HCV: 405	NA	HIV: HR 1·88 (0·27–13·03);aHR 1·96 (0·31–12·27) HCV: HR 1·69 (1·1–2·6);aHR 1·45 (0·92–2·28)	Recent incarceration (past 6 months), MMT coverage, crack injecting (past 6 months)	HIV: 8 HCV: 8
Dumchev (unpublished estimate)	2013–15	Ukraine	Prospective cohort^51^	Current homelessness	HIV and HCV	HIV: 2157 HCV: 2157	HIV: 1·8 HCV: 21·5	HIV: IRR 1·16 (0·07–18·86) HCV: HR 1·80 (0·80–4·07);aHR 1·57 (0·69–3·54)	Ever been in prison, age (continuous), injecting drug use duration (continuous)	HIV: 6 HCV: 6
Hagan et al (2001)^41^	1994–97	Seattle, WA, USA	Prospective cohort	Homelessness in the past 12 months	HCV	317	16·7	RR 1·08 (0·59–1·97)	NA	5
Hagan et al (2010)^52^	2002–04	Baltimore, MA; Chicago, IL; Los Angeles, CA; New York, NY; and Seattle, WA USA	Prospective cohort	Homelessness in the past 6 months	HCV	483	17·2	HR 0·93 (0·68–2·29)	NA	5
Hayashi (unpublished estimate)	1996–2016	Vancouver, BC, Canada	Prospective cohort^32^	Homelessness in the past 6 months	HIV and HCV	HIV: 1763 HCV: 387	NA	HIV: HR 0·78 (0·54–1·14);aHR 0·73 (0·50–1·06);HCV: HR 1·57 (1·11–2·22);aHR 1·62 (1·14–2·29)	Recent incarceration (past 6 months), MMT coverage, crack injecting (past 6 months)	HIV: 8 HCV: 9
Hope et al (2018)^33^	2011–13	England, Wales, and Northern Ireland, UK	Cross-sectional	Homelessness in the past 12 months	HCV	2816	12·3	RR 1·40 (1·02–1·92)	NA	3
Hope (unpublished estimate)	2006–09	Birmingham, Bristol, Glasgow, and Leeds, UK	Cross-sectional^53^	Homelessness in the past 12 months	HCV	1247	16·9	RR 1·85 (0·72–4·73);aRR 1·62 (0·55–4·56)	Recent incarceration (past 12 months), current OST status, cocaine use, duration of injecting	6
Judd (unpublished estimate)	2001–03	London and Brighton, UK	Prospective cohort^54^	Unstable housing in the past 12 months	HIV and HCV	HIV: 263 HCV: 149	HIV: 3·5 HCV: 39·7	HIV: HR 0·94 (0·23–3·76) HCV: HR 1·53 (0·84–2·77)	NA	HIV: 5 HCV: 5
Kåberg (unpublished estimate)	1987–2020	Stockholm, Sweden	Retrospective cohort^55^	Homelessness in the past 3 months	HCV	832	17·5	HR 2·12 (1·62–2·78)	NA	5
Kral et al (2001)^56^	1986–98	San Francisco, CA, USA	Case control	Current homelessness	HIV	6115	1·2	OR 1·24 (0·71–2·17)	NA	4
Kurth (unpublished estimate)	2012–15	Nairobi and Coastal region of Kenya	Retrospective cohort^57^	Current homelessness	HIV	978	2·6	IRR 3·45 (1·48–7·62)	NA	5
Lucidarme et al (2004)^58^	1999–2001	Northern and eastern France	Prospective cohort	Unstable housing in the past 3 months	HCV	165	9·0	IRR 2·20 (0·51–7·22)	NA	5
Maher (unpublished estimate)	1999–2002	New South Wales, Australia	Prospective cohort^59^	Unstable housing in the past 6 months	HCV	258	26·1	HR 1·01 (0·31–3·23)	NA	6
Maher (unpublished estimate)	2008–14	Sydney, NSW, Australia	Prospective cohort^60^	Unstable housing in the past 6 months	HCV	169	6·6	HR 1·17 (0·58–2·36)	NA	7
Mehta (unpublished estimate)	1993–2019	Baltimore, MD, USA	Prospective cohort^61^	Homelessness in the past 6 months	HIV and HCV	HIV: 2456 HCV: 1731	HIV: 1·1 HCV: 0·9	HIV: IRR 1·58 (1·15–2·17);aIRR 1·16 (0·84–1·60);HCV: IRR 1·74 (1·11–2·73);aIRR 1·66 (1·01–2·74)	Injected cocaine in past 6 months; incarcerated in past 6 months; OST or MAT	HIV: 8 HCV:9
Mehta (unpublished estimate)	2013	India	Cross-sectional^62^	Current homelessness	HIV	9440	5·2	IRR 1·56 (0·90–2·70);aIRR 1·52 (0·88–2·63)	Injected stimulants in past 6 months, participated in OST programme in past 6 months, incarcerated in past 6 months	6
Morris (unpublished estimate)	2000–19	San Francisco, CA, USA	Prospective cohort^63^	Homelessness in the past 3 months	HCV	712	24·9	HR 1·95 (1·44–2·64);aHR 1·65 (1·21–2·25)	Gender, age, injecting frequency, recent unsafe injecting behaviours, number of injecting partners	5
Niccolai et al (2011)^64^	2005–08	St Petersburg, Russia	Cross-sectional	Homelessness in the past 12 months	HIV	438	Estimate 1: 18·7* estimate 2: 20·0*	RR 0·70 (0·33–1·52)	NA	4
Palmateer et al (2014)^65^	2008–12	Scotland, UK	Cross-sectional	Homelessness in the past 6 months	HCV	7951	10·0	RR 3·80 (2·20–6·57)	NA	5
Sacks-Davis (unpublished estimate)	2005–10	Melbourne, VIC, Australia	Prospective cohort^66^	Current unstable housing	HCV	89	15·4	HR 1·63 (0·72–3·70):aHR 1·58 (0·66–3·79)	OST (any pharmacotherapy in the past 3 months), type of infection (primary, reinfection), correlation within individuals	6
Samo et al (2013)^67^	2009–11	Karachi, Pakistan	Prospective cohort	Current homelessness	HIV	474	12·4	IRR 1·70 (1·20–2·50);aIRR 1·70 (1·10–2·50)	Sharing of syringes, non-Muslim religion, daily frequency of injecting drugs, source of registration (registered with drop-in centres through outreach compared with other methods), physical disability, monthly income and sources of syringes or needles	5
Schulkind et al (2019)^68^	2012–16	Dundee, UK	Prospective cohort	Current unstable housing	HCV	94	21·5	IRR: 0·42 (0·06–3·23)	NA	6
Spittal et al (2012)^69^	2003–09	Vancouver, BC, Canada	Prospective cohort	Homelessness in the past 6 months	HCV	148	11·6	HR 1·26 (0·83–1·90)	NA	5
Strathdee (unpublished estimate)	2006–10	Tijuana, Mexico	Prospective cohort^70^	Unstable housing in the past 6 months	HIV	812	0·9	HR 1·50 (0·55–4·07)	NA	7
Strathdee (unpublished estimate)	2011–20	Tijuana, Mexico	Prospective cohort^71^	Unstable housing in the past 6 months	HIV	472	2·5	HR 2·10 (1·13–3·90)	NA	5
Sypsa et al (2017)^31^	2012–13	Athens, Greece	Retrospective cohort	Current homelessness	HIV	3320	4·5	HR 1·75 (1·30–2·36);aHR 1·96 (0·98–3·85)	Age, sex, country of origin, history of any imprisonment, size of participant's network of PWID, currently on OST programme, main substance of use, injecting drug use in past 1 month, frequency of injecting drug use, sharing syringes, use of drugs divided with a syringe that someone else had already used for injection	8
Sypsa (unpublished estimate)	2012–13	Athens, Greece	Retrospective cohort^31^	Current homelessness	HCV	63	64·5	IRR 2·31 (0·86–6·19)	NA	5
Thorpe et al (2002)^72^	1997–99	Chicago, IL, USA	Prospective cohort	Homelessness in the past 6 months	HCV	353	10·0	HR 0·76 (0·31–1·86);aHR 0·63 (0·25–1·58)	Injection related risk exposures (sharing cookers, sharing cotton filters, sharing rinse water, sharing syringes), demographic covariates (high-school diploma, suburban residence), drug use covariates (daily injection in the past 6 months, cocaine injection in the past 6 months)	6
Todd (unpublished estimate)	2007–09	Kabul, Afghanistan	Prospective cohort^73^	Homelessness in the past 6 months	HIV and HCV	HIV: 316 HCV: 191	HIV: 1·5 HCV: 40·4	HIV: HR 0·45 (0·05–3·91) HCV: HR 0·76 (0·45–1·29)	NA	HIV: 6 HCV: 6
Valencia (unpublished estimate)	2003–16	Madrid, Spain	Prospective cohort^74^	Current homelessness	HCV	127	60·4	HR 3·82 (0·80–16·9);aHR 4·90 (1·07–23·1)	Crack injecting, OST exposure	7
Vallejo et al (2015)^75^	2001–06	Barcelona, Madrid, and Valencia, Spain	Prospective cohort	Unstable housing in the past 12 months	HCV	513	39·8	IRR 1·71 (0·9–3·25)	NA	5
Van Santen (unpublished estimate)	1989–2014	Amsterdam, Netherlands	Prospective cohort^76^	Homelessness in the past 6 months	HIV and HCV	HIV: 690 HCV: 174	HIV: 1·2 HCV:3·9	HIV: HR 2·02 (1·01–4·02);aHR 2·02 (1·01–4·01) HCV: HR 2·95 (1·39–6·23);aHR 3·04 (1·42–6·52)	Methadone dosing (no methadone *vs* <60 mg/day *vs* ≥60 mg/day)	HIV: 6 HCV: 6
Wijnand (unpublished estimate)	2011–19	Melbourne, VIC, Australia	Prospective cohort^77^	Current homelessness or unstable housing	HCV	125	5·7	Homelessness: HR 1·18 (0·16–8·86);aHR 1·09 (0·14–8·59) Unstable housing: HR 1·19 (0·41–3·48);aHR 1·21 (0·40–3·68)	Methamphetamine (ice, crystal, or shabu) injected in the past month, OST	8

Data in parentheses are 95% CIs. For unpublished estimates, studies are listed by the name of the investigator who provided the data or the unpublished estimate and we cited the most recently published article that was based on the same cohort. Unpublished estimates are those that have been calculated from raw data for this study and have not been presented in previously published papers. aHR=adjusted hazard ratio. aIRR=adjusted incidence rate ratio. aRR=adjusted relative risk. BED EIA=BED capture enzyme immunoassay. HCV=hepatitis C virus. HR=unadjusted hazard ratio. IRR=unadjusted incidence rate ratio. MAT=medication-associated treatment. MMT=methadone maintenance treatment. NA=not applicable. NEP=needle exchange programme. OAT=opioid agonist therapy. OST=opioid substitution treatment. RR=unadjusted relative risk.

* Two estimates for HIV incidence were available because of use of two different formulas for incidence estimation to adjust for misclassification due to sensitivity and specificity characteristics of the BED EIA.

**Figure 2:**
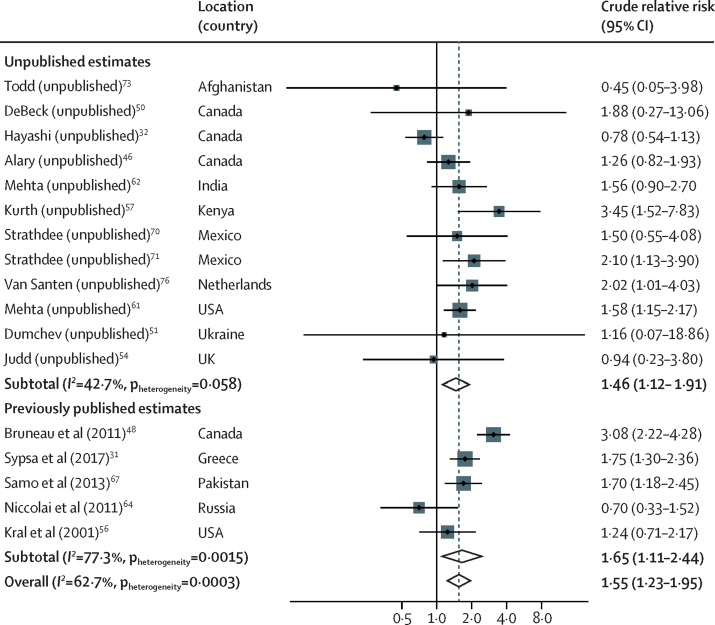
Meta-analysis of studies showing the unadjusted effect of recent homelessness or unstable housing on risk of HIV acquisition in PWID compared with PWID who are not homeless or are stable housed, by publication status For unpublished estimate studies, we listed the studies by the principal investigator who supplied the data and referenced the most recently published article that was based on the same cohort. Unpublished estimates are those that have been calculated from raw data for this study and have not been presented in previously published studies. PWID=people who inject drugs.

17 studies provided 26 estimates (of which 18 were unpublished estimates) for the association between recent homelessness or unstable housing and risk of HIV acquisition. 17 estimates were unadjusted estimates and nine were adjusted estimates (table 1). In the unadjusted analysis, compared with PWID who were not recently homeless or were stably housed, recent homelessness or unstable housing was associated with a crude RR (cRR) for HIV acquisition of 1·55 (95% CI 1·23–1·95; p=0·0002) with substantial between-study heterogeneity (*I*^2^=62·7%; p=0·0003; table 2; figure 2). In the sensitivity analyses, the pooled unadjusted estimate from published estimates was similar (cRR 1·65 [95% CI 1·11–2·44]; p=0·012) to the pooled unadjusted estimate of those calculated from raw data (1·46 [1·12–1·91]; p=0·0048; table 2; figure 2). The risk was reduced slightly when adjusted estimates were pooled (adjusted RR 1·39 [1·06–1·84]; p=0·019). The direction of association was consistent and similar in all other sensitivity analyses, except when considering the association between recent unstable housing and risk of HIV acquisition, for which the effect size was larger (cRR 1·82 [1·13–2·95]; p=0·014; table 2). Additionally, when pooling studies with at least 90% of participants with recent injecting the association was no longer significant. In the subgroup and meta-regression analyses, we found no evidence that the effect of recent homelessness or unstable housing on HIV acquisition risk differed by region, baseline characteristics of study participants, or study characteristics (appendix pp 11–12).

**Table 2:** Sensitivity analysis of the effect of recent homelessness or unstable housing on risk of HIV or HCV acquisition in PWID compared with PWID who are not homeless or are stably housed

	**Number of estimates**	**Effect size (95% CI)**	**p value**	***I*^2^**	***p*_heterogeneity_**
**Effect on risk of HIV acquisition**					
Unadjusted effect estimates	17	1·55 (1·23–1·95)	0·0002	62·7	0·0003
Adjusted effect estimates	9	1·39 (1·06–1·84)	0·019	65·5	0·0031
Published unadjusted estimates	5	1·65 (1·11–2·44)	0·012	77·3	0·0015
Unpublished unadjusted estimates*	12	1·46 (1·12–1·91)	0·0048	42·7	0·058
Only longitudinal studies	15	1·62 (1·27–2·07)	0·0001	63·4	0·0005
Only studies with hazard ratios	10	1·56 (1·08–2·25)	0·018	73·5	<0·0001
Only studies with at least 90% of participants injecting recently	11	1·44 (0·97–2·13)	0·068	75·4	0·0001
Only studies at low-to-moderate risk of bias	10	1·56 (1·14–2·12)	0·0052	72·6	0·0001
Estimate for homelessness	12	1·44 (1·13–1·83)	0·0036	52·7	0·016
Estimate for unstable housing	5	1·82 (1·13–2·95)	0·014	67·8	0·014
**Effect on risk of HCV acquisition**†					
Unadjusted effect estimates	28	1·65 (1·44–1·89)	<0·0001	44·8	0·0060
Adjusted effect estimates	14	1·64 (1·43–1·89)	<0·0001	9·6	0·35
Published unadjusted estimates	11	1·61 (1·18–2·19)	0·0029	66·9	0·0008
Unpublished unadjusted estimates*	17	1·69 (1·49–1·92)	<0·0001	14·2	0·29
Only longitudinal studies	25	1·61 (1·40–1·86)	<0·0001	38·6	0·027
Only studies with hazard ratios	18	1·59 (1·36–1·86)	<0·0001	45·6	0·019
Only studies with at least 90% of participants injecting recently	20	1·54 (1·32–1·80)	<0·0001	39·0	0·039
Only studies at low-to-moderate risk of bias	19	1·67 (1·37–2·03)	<0·0001	52·4	0·0042
Estimate for homelessness	19	1·66 (1·37–2·00)	<0·0001	55·3	0·0018
Estimate for unstable housing	10	1·72 (1·48–1·99)	<0·0001	0·0	0·47

HCV=hepatitis C virus. PWID=people who inject drugs.

* Unpublished estimates are those that have been calculated from raw data for the current study and have not been presented in previously published papers.

† One study (unpublished estimate, provided by Wijnand and colleagues) provided estimates for both unstable housing and homelessness. For all sensitivity analyses, the effect of unstable housing from this study was used, except when pooling estimates specifically for homelessness or unstable housing. Hence, the number of studies for these two sensitivity analyses add up to 29.

**Figure 3:**
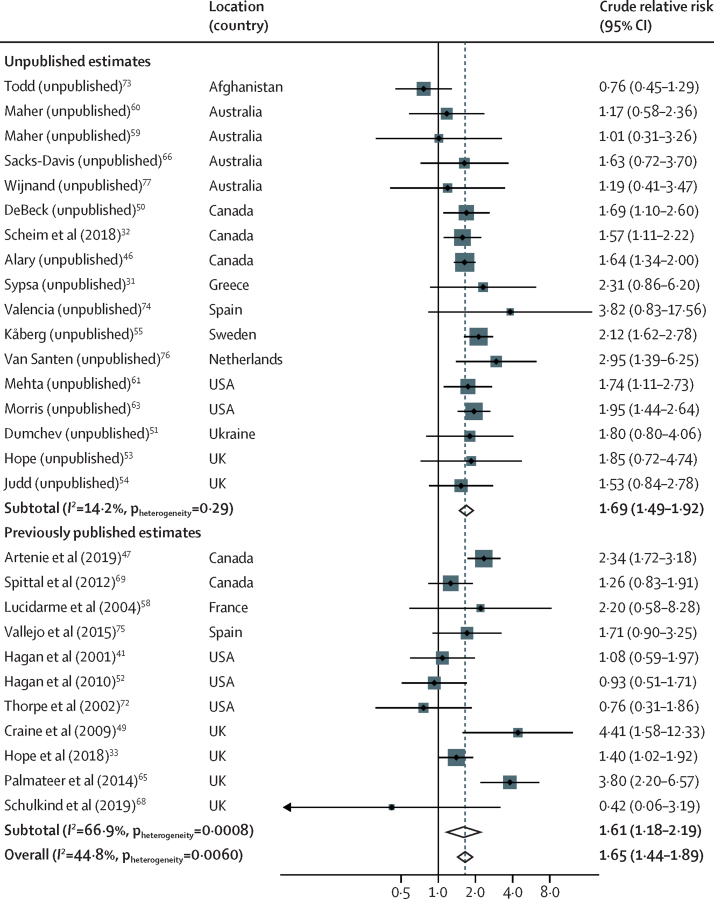
Meta-analysis of studies showing unadjusted effect of recent homelessness or unstable housing on risk of HCV acquisition in PWID compared with PWID who are not homeless or are stably housed, by publication status For unpublished estimate studies, we listed the studies by the principal investigator who supplied the data and referenced the most recently published article that was based on the same cohort. Unpublished estimates are those that have been calculated from raw data for this study and have not been presented in previously published studies. HCV=hepatitis C virus. PWID=people who inject drugs.

28 studies provided 44 estimates (of which 30 were unpublished) of the association between homelessness or unstable housing and risk of HCV acquisition. One study (unpublished estimate, provided by Wijnand and colleagues; table 1) provided unadjusted and adjusted effect estimates for both homelessness and unstable housing, with the definition of unstable housing encompassing homelessness. For all analyses, we used the estimate for unstable housing, except for the sensitivity analysis where effect estimates for homelessness and unstable housing were pooled separately. Therefore, in the main analyses, we included 28 unadjusted and 14 adjusted estimates (table 1). In the unadjusted analysis, compared with PWID who were not homeless or were stably housed, recent homelessness or unstable housing was associated with a cRR for HCV acquisition of 1·65 (95% CI 1·44–1·89; p<0·0001) with moderate between-study heterogeneity (*I*^2^= 44·8%, p=0·0060; table 2; figure 3). In sensitivity analyses, the pooled unadjusted estimate from published estimates was similar (cRR 1·61 [1·18–2·19; p=0·0029) to the pooled unadjusted estimate of those calculated from raw data (1·69 [1·49–1·92]; p<0·0001; table 2; figure 3). The risk was similar when adjusted estimates were pooled (adjusted RR 1·64 [1·43–1·89]; p<0·0001; table 2). The association was similar across all other sensitivity analyses (table 2). The subgroup and meta-regression analyses suggest that the association between recent homelessness or unstable housing and HCV acquisition risk was higher in studies done in Europe than elsewhere (North America, Australasia, and south and central Asia), higher in studies with greater baseline OST coverage, and higher in studies spanning longer time periods (appendix pp 12–14).

We found no evidence of asymmetry in funnel plots for either of the two summary measures by visual inspection or Egger's test (p=0·51 for both HIV and HCV; appendix pp 16–17). Overall, the risk of bias score based on the Newcastle-Ottawa Scale varied from 4 points (three studies) to 9 points (two studies), and the median score did not differ for estimates of HIV and HCV studies, both being 6, indicating a low-to-moderate risk of bias (table 1; appendix pp 29–31).

## Discussion

We found strong evidence of an increased risk of HIV and HCV acquisition among PWID who are exposed to recent homelessness or unstable housing compared with PWID who are not homeless or are stably housed. Across all included studies, recent homelessness or unstable housing was associated with a 1·55 times greater risk of HIV acquisition and a 1·65 times greater risk of HCV acquisition. Although our estimates remained largely consistent in most sensitivity analyses, the pooled adjusted estimates indicated a slightly lower effect for HIV (RR 1·39) but not HCV (RR 1·64).

The association between recent homelessness or unstable housing and risk of HIV acquisition did not vary according to any of the factors considered, including participant and study characteristics and geographical or economic region. Conversely, the association with risk of HCV acquisition was greater in studies done in Europe, studies done in regions of higher OST coverage, and in longer studies. The reasons for these associations are unclear. Possibly, compared with North America and Australasia, PWID who are homeless or unstably housed in Europe are less likely to attend harm-reduction services, and thus are more likely to engage in high-risk behaviours. Alternatively, the definition of homelessness or unstable housing adopted in studies done in Europe might have been more likely to capture those at higher risk of HCV infection than studies done outside of Europe. Overall, although our findings could reflect true differences, they should be interpreted with caution. Some covariates had many missing values because they were not systematically reported in all studies, leading to sparse data across some categories. Also, some differences might be due to confounding, at least partly, given that the high number of missing values precluded multivariable meta-regression analyses.

Our finding of greater risk of HIV and HCV infection among PWID who experience housing instability than among PWID who are not homeless or who are stably housed can be attributed to increased injection and high-risk sexual behaviours in this population, as previously reported.^22^, ^23^, ^44^, ^78^, ^79^ These behaviours are probably the result of broader health and social factors and difficult socioeconomic circumstances experienced by homeless and unstably housed PWID, including inadequate access to harm-reduction services,^25^, ^26^, ^30^, ^40^, ^80^ mental health disorders,^44^ incarceration,^23^, ^24^, ^44^ unemployment,^44^ and food insecurity.^81^ Overall, our findings align with the syndemic framework for conceptualising health outcomes, which suggests that overlapping biosocial problems do more than just cluster together but exacerbate health inequalities.^82^ Both homelessness and drug dependence carry stigma, discrimination, and structural inequalities, and together can amplify susceptibility to adverse health outcomes. For example, in studies exploring the perceptions of homeless PWID, the stress surrounding their precarious living circumstances and the pervasiveness of drugs in the social environment were found to considerably amplify drug use and high-risk behaviours.^79^, ^83^, ^84^

Housing First^85^, ^86^, ^87^ is one initiative that aims to reduce homelessness that has been adopted in some high-income countries. This model centres on providing immediate housing to marginalised populations, and is not contingent on them being enrolled into drug treatment services or ceasing drug use. Additional support for those with mental health and substance use disorders is also offered. Although the effectiveness of this initiative on HIV and HCV transmission and injecting-related risk behaviours has yet to be determined, it has been shown to have a positive impact on mental health and substance use, social integration, housing stability, quality of life, and involvement with the criminal justice system.^87^, ^88^ Other housing interventions can also reduce high-risk sexual behaviours and improve treatment outcomes among homeless populations living with HIV,^89^ but few studies have considered homeless PWID.

Despite evidence of their benefit, housing initiatives are not well established in most high-income countries,^87^ and even less so in low-income and middle-income countries. However, during the ongoing COVID-19 pandemic, numerous countries (including the UK, France, Australia, and the USA) rapidly escalated their efforts to provide safe and secure housing to homeless or unstably housed individuals.^90^, ^91^, ^92^ In England, UK, these efforts have not only potentially reduced COVID-19 outbreaks in this group,^93^ but has also resulted in increased linkage to care with opportunities to address long-standing health issues, such as testing and treatment for tuberculosis, HIV, and HCV.^94^ Comprehensive efforts like these that provide housing alongside harm-reduction programmes and access to HIV and HCV treatment for those who are infected are needed to achieve meaningful reductions in transmission. Expanding access to treatment among homeless PWID should be prioritised given the unique barriers faced by this group,^95^, ^96^ despite the availability of effective and cost-effective HIV and HCV testing and treatment interventions.^97^, ^98^, ^99^

A strength of our study is that it includes 48 unpublished estimates, increasing the number of included estimates from 22 to 70, so minimising the risk of publication bias. Nonetheless, our study had several limitations. First, because our review involved observational studies, we cannot rule out unmeasured confounding. Although our estimates remained consistent after adjustment for OST exposure, history of incarceration, and stimulant injecting, and we found a low-to-moderate risk of bias across studies, other factors might have confounded the association (eg, ethnicity, severity of addiction, or mental health disorders, which are rarely measured in these studies). Second, selection bias is another limitation of longitudinal observational studies because PWID who are homeless or unstably housed might have increased attrition. If participants who were lost to follow-up had a higher risk of acquisition than those who remained in follow-up, we might have underestimated the true associations between homelessness or unstable housing and risk of HIV and HCV acquisition. Importantly, we assessed selection bias using the Newcastle-Ottawa Scale and our estimates remained consistent when pooling studies at low-to-moderate risk of bias. Third, differences across studies in the measurement of homelessness and unstable housing could have introduced bias in the pooled estimates. Our sensitivity and meta-regression analyses revealed some non-significant differences between estimates that defined exposure using different timeframes (eg, past year *vs* past 6 months or currently) or based on whether participants were homeless or unstably housed. Unfortunately, we did not have the statistical power to draw conclusions with regards to these differences. These limitations are likely to be amplified by the absence of a universal definition of homelessness or unstable housing that accounts for differences across sociocultural settings, making the monitoring of homelessness and its effect on health outcomes across different countries and regions difficult. We were also restricted in our ability to examine whether the association between homelessness and unstable housing and HIV or HCV risk varied as a function of other factors, such as coverage of needle and syringe provision or antiretroviral therapy. As mentioned, meta-regression analyses were underpowered to detect significant differences due to the high proportion of missing data. Fourth, data from low-income and middle-income countries was scarce, with only nine studies being available from such settings for this systematic review and meta-analysis (seven HIV studies and two HCV studies). Further studies are needed from low-income and middle-income countries to enable us to draw more generalisable conclusions. Finally, although details of the methods were prespecified and documented in a protocol, we were unable to register our protocol as intended due to changes in the PROSPERO eligibility criteria during the conduct of our study.

In summary, our study provides strong evidence that current or recent homelessness and unstable housing is associated with increased risk of HIV and HCV acquisition among PWID. These findings frame housing instability as an important driver of HIV and HCV transmission among PWID and call for intensified efforts to assess and implement housing initiatives and targeted prevention services that are tailored to the needs of this marginalised population. To help PWID attain and maintain housing stability, integrated strategies that address their competing health and social concerns are urgently needed. Great changes, albeit only the temporary provision of housing, achieved during the COVID-19 pandemic have shown that such strategies are possible when there is sufficient political will.

## Data sharing

Extracted data sheets will be made available immediately after publication of this Article. These data sheets will be shared with researchers who provide a methodologically sound proposal approved by JS and PV. Proposals should be directed to jack.stone@bristol.ac.uk and Peter.vickerman@bristol.ac.uk; requesters will need to sign a data access agreement.

## Declaration of interests

HF reports honoraria from MSD outside of the submitted work. JA reports grants from National Institutes of Health (NIH) during the conduct of the study. SSS reports grants from NIH during the conduct of the study, and grants from Gilead Sciences and Abbot Diagnostics outside of the submitted work. VS reports grants, personal fees, and non-financial support from Gilead Sciences and AbbVie and personal fees from Janssen outside of the submitted work. JGW reports grants from Gilead Sciences outside of the submitted work. All other authors declare no competing interests.
