# Supplementary material for: Homelessness, unstable housing, and risk of HIV and hepatitis C virus acquisition among people who inject drugs: a systematic review and meta-analysis
Source: Lancet Public Health. 2021 Mar 26;6(5):e309–23. doi: 10.1016/S2468-2667(21)00013-X (PMC8097637; doi:10.1016/S2468-2667(21)00013-X)
Supplement: Supplementary appendix [file mmc1.pdf]

THE LANCET
Public Health

Supplementary appendix

This appendix formed part of the original submission and has been peer reviewed. We post it as supplied by the authors.

Supplement to: Arum C, Fraser H, Artenie AA, et al. Homelessness, unstable housing, and risk of HIV and hepatitis C virus acquisition among people who inject drugs: a systematic review and meta-analysis. *Lancet Public Health* 2021; published online March 26. http://dx.doi.org/10.1016/S2468-2667(21)00013-X.

Appendix table 1: PRISMA checklist

SECTION/TOPIC	#	CHECKLIST ITEM	REPORTED ON PAGE #
**TITLE**			
**Title**	1	Identify the report as a systematic review, meta-analysis, or both.	1
**ABSTRACT**			
**Structured summary**	2	Provide a structured summary including, as applicable: background; objectives; data sources; study eligibility criteria, participants, and interventions; study appraisal and synthesis methods; results; limitations; conclusions and implications of key findings; systematic review registration number	3
**INTRODUCTION**			
**Rationale**	3	Describe the rationale for the review in the context of what is already known.	6
**Objectives**	4	Provide an explicit statement of questions being addressed with reference to participants, interventions, comparisons, outcomes, and study design (PICOS)	6
**METHODS**			
**Protocol and registration**	5	Indicate if a review protocol exists, if and where it can be accessed (e.g., Web address), and, if available, provide registration information including registration number.	7, Appendix page 3
**Eligibility criteria**	6	Specify study characteristics (e.g., PICOS, length of follow-up) and report characteristics (e.g., years considered, language, publication status) used as criteria for eligibility, giving rationale.	7
**Information sources**	7	Describe all information sources (e.g., databases with dates of coverage, contact with study authors to identify additional studies) in the search and date last searched.	7
**Search**	8	Present full electronic search strategy for at least one database, including any limits used, such that it could be repeated.	Appendix page 7
**Study selection**	9	State the process for selecting studies (i.e., screening, eligibility, included in systematic review, and, if applicable, included in the meta-analysis).	7
**Data collection process**	10	Describe method of data extraction from reports (e.g., piloted forms, independently, in duplicate) and any processes for obtaining and confirming data from investigators.	7,8
**Data items**	11	List and define all variables for which data were sought (e.g., PICOS, funding sources) and any assumptions and simplifications made.	8, Appendix page 8
**Risk of bias in individual studies**	12	Describe methods used for assessing risk of bias of individual studies (including specification of whether this was done at the study or outcome level), and how this information is to be used in any data synthesis.	7-9
**Summary estimates**	13	State the principal summary measures (e.g., risk ratio, difference in means).	7-9
**Synthesis of results**	14	Describe the methods of handling data and combining results of studies, if done, including measures of consistency (e.g., I2) for each meta-analysis.	9
**Risk of bias across studies**	15	Specify any assessment of risk of bias that may affect the cumulative evidence (e.g., publication bias, selective reporting within studies).	8,9
**Additional analysis**	16	Describe methods of additional analyses (e.g., sensitivity or subgroup analyses, meta-regression), if done, indicating which were pre-specified.	8,9

**RESULTS**			
**Study selection**	17	Give numbers of studies screened, assessed for eligibility, and included in the review, with reasons for exclusions at each stage, ideally with a flow diagram.	9,20
**Study characteristics**	18	For each study, present characteristics for which data were extracted (e.g., study size, PICOS, follow-up period) and provide the citations.	9,21-27
**Risk of bias within studies**	19	Present data on risk of bias of each study and, if available, any outcome level assessment (see item 12).	9, Appendix page 29-31
**Results of individual studies**	20	For all outcomes considered (benefits or harms), present, for each study: (a) simple summary data for each intervention group (b) effect estimates and confidence intervals, ideally with a forest plot.	9-11,28-29 Appendix page 11-19
**Synthesis of results**	21	Present results of each meta-analysis done, including confidence intervals and measures of consistency.	9-11, Appendix page 11-19
**Risk of bias across studies**	22	Present results of any assessment of risk of bias across studies (see Item 15).	9-11,30 Appendix page 29-31
**Additional analysis**	23	Give results of additional analyses, if done (e.g., sensitivity or subgroup analyses, meta-regression [see Item 16]).	9-11, Appendix page 11-19
**DISCUSSION**			
**Summary of evidence**	24	Summarize the main findings including the strength of evidence for each main outcome; consider their relevance to key groups (e.g., healthcare providers, users, and policy makers).	11,12
**Limitations**	25	Discuss limitations at study and outcome level (e.g., risk of bias), and at review-level (e.g., incomplete retrieval of identified research, reporting bias).	11,12
**Conclusions**	26	Provide a general interpretation of the results in the context of other evidence, and implications for future research.	13,14
**FUNDING**			
**Funding**	27	Describe sources of funding for the systematic review and other support (e.g., supply of data); role of funders for the systematic review.	2,9

PROTOCOL

Effect of homelessness and unstable housing on the risk of HIV and HCV acquisition among people who inject drugs: Protocol for a systematic review and meta-analysis

Background

Morbidity attributable to HIV and HCV has been on the rise globally over the past few decades.[1] Injecting drug use is recognized as a significant risk factor for acquisition of HIV and HCV infection and has been associated with a substantial proportion of the global burden of these infections.[2,3] Globally, out of the 15·6 million PWID aged 15 – 64 years, an estimated 3·4 million (21.7%) have experienced homelessness or unstable housing within the past year.[3]

Homelessness and other forms of unstable housing have been consistently associated with injecting drug use as well as with other poor health outcomes including higher burden of infectious diseases such as HIV, hepatitis B, hepatitis C, and latent tuberculosis infection, deaths from injury, poisoning and other external causes, poor mental health, cardiovascular conditions such as coronary heart disease, and respiratory conditions.[3-6]

Several studies have reported homelessness and unstable housing as part of the micro-environmental factors that potentiate the risk of acquisition of HIV infection among people who inject drugs (PWID).[7-13] Recent evidence also suggests that incarceration history and homelessness/unstable housing may act synergistically in driving the elevated acquisition risk of HIV and hepatitis C among PWID after release from prison, thereby highlighting the important effects homelessness and unstable housing can have in this population.[14] However, to our knowledge, no systematic review has been undertaken to synthesise the effects of homelessness of HIV or HCV acquisition risk among PWID. Therefore, in order to improve the evidence base, inform policy decisions on inclusion health[15] and enhance HIV and HCV prevention strategies, a systematic review of this evidence would be of significance.

Aim/Objectives

This systematic review will aim to synthesize the effect of current or recent homelessness and unstable housing on the risk of HIV and HCV acquisition among PWID. Specific objectives will aim to clarify whether there is an association between:

1. Current or recent homelessness/unstable housing and acquisition risk/incidence of HIV among PWID.
2. Current or recent homelessness/unstable housing and acquisition risk/incidence of HCV among PWID.

Methods

Eligibility criteria

Population: People who inject drugs.

Study setting: Community setting

Outcomes: Outcome data on association between homelessness and incidence of HIV or HCV. Studies of incidence of primary HCV infection or HCV reinfection will be included. Studies where incidence is estimated based on recent infection determined by serological tests such as testing HCV Ab negative and HCV RNA positive, HCV avidity, or BED Assay for HIV will also be included.

Study designs: All study designs will be included and original/primary studies only.

Language: No language restriction.

Information sources/ Search strategy

An existing database of HIV and HCV incidence studies published from Jan 1, 2000 to June 13, 2017 was updated. This database was used for a previous review on incarceration history and risk of HIV and hepatitis C virus acquisition among PWID.[14] Without language restriction and limited to studies published after June 13, 2017, a systematic literature search of MEDLINE, Embase, and PsycINFO databases was conducted using similar strategy developed for the incarceration review.

Data management

Endnote X9 will be used to catalogue search results. These will be de-duplicated using the ‘find duplicate’ feature. Titles and abstracts will be screened based on the inclusion criteria. Full text of relevant references will be obtained and for studies that could not be explicitly excluded based on their titles or abstracts for a more thorough screening. The full text will be used to determine the eligibility for inclusion and for exclusion of studies, with clear reasons documented. In order to facilitate the creation of a PRISMA flow diagram, the ‘group’ functionality will be used to track results from each stage of the selection process. The selected list of references to be included in the review will be further stratified into those relating to HIV or HCV or both.

Selection process

All references will be screened by one author through to the full text stage. In addition, 10% of the reference will be screened by multiple authors through to the full text stage. In a case of inconsistences between the lists of accepted references, the remaining references will be double screened. Any ensuing discord that cannot be resolved by discussion will be adjudicated by a third author.

Data extraction process

Data from selected references will be extracted using Microsoft Excel 2016. This will be done by one author and will be checked for accuracy by a second author with any discord not resolvable by discussion adjudicated by a third. For studies on incidence of HIV or HCV among PWID that do not report on the outcome of interests (effect of homelessness or unstable housing on HIV or HCV incidence) or with ambiguous effect estimates, the authors will be contacted to request unpublished effect estimates or to resolve any ambiguities.

Data items

Data will be extracted on:

- Lead author, review title or unique identifier and date
- Study design (including sampling methods, participants, and attrition rate)
- Study location
- Study setting
- Study period (over which follow-ups are performed)
- Baseline sample size
- Study inclusion and exclusion criteria (including definition of PWID, e.g. injecting in the last 30 days)
- Attrition rate with comments on attrition.
- Methods of assessment of HIV/HCV infection and HCV re-infection
- Age of participants (median/mean)
- Sex of participants (proportion of males/ females)
- Duration of injecting of participants (mean/median)
- Incarceration history (proportion ever incarcerated)
- OST coverage
- Proportion of homeless participants
- Definition of homelessness
- Duration of follow up, overall and by housing status
- Outcome measure (HCV or HIV seroconversion); overall and by housing status
- Unadjusted and adjusted effect size (incidence rate ratio (IRR); odds ratio (OR); hazard ratio (HR); and precision (e.g. 95% confidence interval (CI)).
- Confounding factors used to adjust effect estimates
- Background HIV/HCV prevalence.
- Risk of bias scores.

Risk of Bias Assessment

The risk of bias will be assessed using the Newcastle-Ottawa Scale (NOS scale) for assessing quality of non-randomized studies. The scale is designed to award a maximum of 9 stars to any study in relation to selection of participants, comparability of cohorts based on design and analysis, and methods of ascertainment of study outcomes. Confounding factors such as Opioid Substitution treatment (OST), stimulant injecting and recent incarceration will be adjusted for.

Data Synthesis

A descriptive summary of findings from selected studies will be included in the review bearing in mind that there may be considerable heterogeneity between studies. For studies with sufficiently similar effect estimates, a meta-analysis will be performed on the crude and adjusted effects of homelessness and unstable housing on incidence of HIV/HCV infection. A random effect meta-analysis will be adopted considering the expectation of some degree of heterogeneity between studies.

Assessment of Heterogeneity

In order to explore whether observed differences in result from the studies are compatible with chance alone, heterogeneity will be examined through inspection of the forest plot, by a Chi^2 test and I-squared statistics. This will further be explored through subgroup analysis and sensitivity analysis.

Assessment of Reporting Biases

To assess publication bias, funnel plots will be used to plot the study effect size against sample size. Funnel plot asymmetry will be assessed by Egger's test.

Subgroup Analysis and Investigation of Heterogeneity

Where enough data are available, subgroup analysis and meta-regression will be undertaken to compare outcomes by:

- Region
- Year of study
- Mean age and duration of injecting
- Proportion of study sample females
- Definition of homelessness
- Proportion of PWID with recent history of incarceration
- OST coverage at baseline
- Publication status – published or unpublished.

All variables found to be significant in the univariable meta-regression will be included in multi-variate analysis if there are enough studies. Similarly, analysis of confounding will be undertaken using subgroup analyses and meta-regression to compare adjusted effect estimates based on whether they were adjusted for:

- Current/recent OST coverage
- Recent history of incarceration
- Current/recent stimulant injecting

Sensitivity analyses

Sensitivity analyses will be performed to assess the impact of including in separate meta-analyses only: studies at low/moderate risk of bias; longitudinal studies; studies reporting hazard ratios; studies with at least 90% recent injectors (injected within last 12 months) at baseline.

References

1. Lozano R, Naghavi M, Foreman K, et al. Global mortality from 235 causes of death for 20 age groups in 1990 and 2010: a systematic analysis for the Global Burden of Disease Study 2010. *Lancet* 2013; 380:2095-128.
2. Degenhardt L, Charlson F, Starnaway J, et al. Estimating the burden of disease attributable to injecting drug use as a risk factor for HIV, hepatitis C, and hepatitis B: findings from the Global Burden of Disease Study 2013. *Lancet Infect Dis* 2016; 16:1385-98.
3. Degenhardt L, Peacock A, Colledge S, et al. Global prevalence of injecting drug use and sociodemographic characteristics and prevalence of HIV, HBV, and HCV in people who inject drugs: a multistage systematic review. *Lancet Glob Health* 2017; 5: e1192-207.
4. Strathdee S A, Hallett T B, Bobrova N, et al. HIV and risk environment for injecting drug users: the past, present, and future. *Lancet* 2010; 376: 268-84.
5. Aldridge R W, Story A, Hwang S W, Nordentoft M, Luchenski S A, Hartwell G, et al. Morbidity and mortality in homeless individuals, prisoners, sex workers, and individuals with substance use disorders in high-income countries: a systematic review and meta-analysis. *Lancet* 2018; 391: 241-50.
6. Aldridge R W, Hayward A C, Hemming S, et al. High prevalence of latent tuberculosis and bloodborne virus infection in a homeless population. *Thorax* 2018; 73: 557-564.

7. Reyes J C, Robles R R, Colon H M, et al. Homelessness and HIV risk behaviours among drug injectors in Puerto Rico. *J Urban Health* 2005; 82: 446-55.
8. Azim T, Chowdhury E I, Reza M, et al. Prevalence of infections, HIV risk behaviours and factors associated with HIV infection among male injecting drug users attending needle/syringe exchange program in Dhaka, Bangladesh. *Subst Use Misuse* 2008; 43: 2124-44.
9. Tyndall M W, Currie S, Spittal P, et al. Intensive injection cocaine use as primary risk factor in the Vancouver HIV-1 epidemic. *AIDS* 2003; 17: 887-93.
10. Zhang Y, Shan H, Trizzino J, et al. HIV incidence, retention rate, and baseline predictors of HIV incidence and retention in a prospective cohort study of injecting drug users in Xinjiang, China. *Int J Infect Dis* 2007; 11: 318-23.
11. Estebanez P E, Russell N K, Aguilar M D, Beland F, Zunzunegui M V. Women, drugs, and HIV/AIDS: results of a multicentre European study. *Int J Epidemiol* 2000; 29: 734-43.
12. Wood E, Kerr T, Marshall B D, et al. Longitudinal community plasma HIV-1 RNA concentrations and incidence of HIV-1 among injecting drug users: prospective cohort study. *BMJ* 2009; 338: b1649.
13. Boileau C, Bruneau J, Al-Nachawati H, Lamothe F, Vincelette J. A prognostic model for HIV seroconversion among injection drug users as a tool for stratification in clinical trials. *J Acquir Immune Defic Syndr* 2005; 39: 489-95.
14. Stone J, Fraser H, Lim A G, et al. Incarceration history and the risk of HIV and hepatitis C virus acquisition among people who inject drugs: a systematic review and meta-analysis. *Lancet Infect Dis* 2018; 18: 1397-409.
15. Luchenski S, Maguire N, Aldridge R W, et al. What Works in inclusion health: overview of effective interventions for marginalised and excluded population*? Lancet* 2018; 391: 266-80.

SEARCH STRATEGY

Medline database search strategy to identify relevant studies. Keywords are listed in regular type and medical subject headings (Mesh) terms are in **Bold.**

1. Hepatitis C OR HCV OR **exp hepatitis C/**
2. HIV OR human immunodeficiency virus OR exp **HIV seropositivity/** OR **exp HIV seroprevalence**/ OR **exp HIV infections**/ OR **exp HIV**/
3. 1 OR 2
4. IDU OR IDUs OR IVDU OR IVDUs OR PWID OR PWIDs
5. (substance* or drug*).mp. [mp=title, abstract, original title, name of substance word, subject heading word, floating sub-heading word, keyword heading word, organism supplementary concept word, protocol supplementary concept word, rare disease supplementary concept word, unique identifier, synonyms]
6. ((substance* or drug*) adj3 (inject* or intravenous)).mp. [mp=title, abstract, original title, name of substance word, subject heading word, floating sub-heading word, keyword heading word, organism supplementary concept word, protocol supplementary concept word, rare disease supplementary concept word, unique identifier, synonyms]
7. ((substance* or drug*) adj3 (abuse* or depend* or use* or misus* or addict*)).mp. [mp=title, abstract, original title, name of substance word, subject heading word, floating sub-heading word, keyword heading word, organism supplementary concept word, protocol supplementary concept word, rare disease supplementary concept word, unique identifier, synonyms]
8. **exp substance abuse, intravenous/**
9. 4 OR 6 OR 7 OR 8
10. Prevalence OR incidence OR epidemiol* OR survey OR rapid assessment OR situation assessment OR situational assessment OR RAR OR cohort OR surveillance OR seroprevalence OR seroincidence OR seroepidemiol* OR seroconv* OR screening OR **exp epidemiologic methods**/ OR **exp epidemiologic studies**/ OR **exp sentinel surveillance**/ OR **exp seroepidemiologic studies**/ OR **exp cohort studies**/ OR **exp cross-sectional studies**/ OR **exp longitudinal studies**/ OR **exp follow-up studies**/ OR **exp prospective studies**/
11. 3 AND 9 AND 10
12. Limit 11: animals/ not (humans/ and animals/)
13. 11 NOT 12
14. **(201706* or 201707* or 201708* or 201709* or 201710* or 201711* or 201712* or 2018* or 2019* or 2020*). dt, ez, ed.**
15. 13 AND 14.

Embase database search strategy to identify relevant studies. Keywords are listed in regular type and Emtree terms in **Bold.**

1. Hepatitis C OR HCV OR **exp hepatitis C**/ OR **exp Hepatitis C virus**/
2. HIV OR human immunodeficiency virus OR **exp human immunodeficiency virus**/ OR **exp human immunodeficiency virus infection**/ OR **exp human immunodeficiency virus prevalence**/
3. 1 OR 2
4. IDU OR IDUs OR IVDU OR IVDUs OR PWID OR PWIDs
5. ((substance* or drug*) adj3 (inject* or intravenous)).mp. [mp=title, abstract, original title, name of substance word, subject heading word, floating sub-heading word, keyword heading word, organism supplementary concept word, protocol supplementary concept word, rare disease supplementary concept word, unique identifier, synonyms]
6. ((substance* or drug*) adj3 (abuse* or depend* or use* or misus* or addict*)).mp. [mp=title, abstract, original title, name of substance word, subject heading word, floating sub-heading word, keyword heading word, organism supplementary concept word, protocol supplementary concept word, rare disease supplementary concept word, unique identifier, synonyms]
7. **exp intravenous drug abuse**/
8. 4 OR 5 OR 6 OR 7
9. Prevalence OR incidence OR epidemiol* OR survey OR rapid assessment OR situation assessment OR situational assessment OR RAR OR cohort OR surveillance OR seroprevalence OR seroincidence OR screening OR **exp seroepidemiology**/ OR **exp seroprevalence**/ OR **exp epidemiology**/ OR **exp prevalence**/ OR **exp epidemiologic data**/ OR **exp incidence**/ OR **exp observational study**/ OR **exp cohort analysis**/
10. 3 AND 8 AND 9
11. Limit 10: animals/ not (humans and animals/)
12. 10 NOT 11
13. **Limit 12 to dc=20170601 – 20200914**

PyscINFO database search strategy to identify relevant studies. Keywords are listed in regular type and Thesaurus terms in **Bold**.

1. Hepatitis C OR HCV OR **exp Hepatitis**/
2. HIV OR human immunodeficiency virus OR **exp HIV**/
3. 1 OR 2
4. IDU OR IDUs OR IVDU OR IVDUs OR PWID OR PWIDs
5. ((substance* or drug*) adj3 (inject* or intravenous)).mp. [mp=title, abstract, original title, name of substance word, subject heading word, floating sub-heading word, keyword heading word, organism supplementary concept word, protocol supplementary concept word, rare disease supplementary concept word, unique identifier, synonyms]
6. ((substance* or drug*) adj3 (abuse* or depend* or use* or misus* or addict*)).mp. [mp=title, abstract, original title, name of substance word, subject heading word, floating sub-heading word, keyword heading word, organism supplementary concept word, protocol supplementary concept word, rare disease supplementary concept word, unique identifier, synonyms]
7. **exp intravenous drug usage**/
8. 4 OR 5 OR 6 OR 7
9. (prevalence or incidence or epidemiol* or survey or rapid assessment or situation assessment or situational assessment or RAR or Cohort or surveillance or seroprevalence or seroincidence or screening).mp. or **exp epidemiology**/ or **exp surveys**/ or **exp cohort analysis**/ or **exp longitudinal studies**/ or **exp follow-up studies**/ or **exp prospective studies**/ [mp=title, abstract, original title, name of substance word, subject heading word, floating sub-heading word, keyword heading word, organism supplementary concept word, protocol supplementary concept word, rare disease supplementary concept word, unique identifier, synonyms]
10. 4 AND 8 AND 9
11. Limit 10: animals/ not (humans/ and animals/)
12. 10 NOT 11
13. **Limit 12 to up=20170601 - 20200914**

DATA EXTRACTION

Data were extracted from included studies using Microsoft Excel 2016 for Windows. Key characteristics that were extracted from individual study included: publication year, study location (city and country), study period (start and end period), study design, cohort name, cohort recruitment sites and methodology, study inclusion and exclusion criteria, duration of follow-up(average), attrition (with comments), methods of assessment of HIV and HCV incident infection and/or reinfection, risk of bias assessment, baseline study characteristics (sample size, proportion who are recent or ever injectors, definition of recent injection, number and proportion of female participants, number and proportion of participants with history of ever been incarcerated, number and proportion of participants with recent history of incarceration, mean/median age and duration of injecting, baseline HIV and HCV prevalence, baseline OST coverage, definition of recent homelessness or unstable housing, proportion homeless at baseline, number and rate of incident infection and number of person-years of follow-up (stratified by housing status: homeless vs not homeless), crude and adjusted effect estimates with 95% uncertainty interval, variables included in the adjusted analysis (with definitions).

Appendix table 2: Details of excluded studies due to duplicate data

Author (Publication Year)	Location	Study period	Cohort name	Sample size	Reported outcome
Aitken et al.[1] (2008)	Melbourne, Australia	2005 - 2007	Network	374	Transient housing
Allen et al.[2] (2012)	Scotland, UK	2008 - 2009	NESI	2629	Recent homelessness (past 6 months)
Bach et al.[3] (2016)	Vancouver, Canada	1996 - 2013	VIDUS & ACCESS	541	Recent homelessness (past 6 months)
Boileau et al.[4]	Montreal, Canada	1992 - 2001	St. Luc Cohort	2444	Unstable housing
Bruneau et al. (2012)[5]	Montreal, Canada	1992 - 2008	St. Luc Cohort	2074	Unstable housing (past 6 months)
Bruneau et al. (2010)[6]	Montreal, Canada	Wave 1: 1988 – 2001<br>Wave 2: 2005 - 2008	St. Luc Cohort	2075	Unstable housing (past 6 months)
Bruneau et al. (2012)[7]	Montreal, Canada	2004 - 2009	St. Luc Cohort	1042	Unstable housing (past 6 months)
Corneil et al. (2006)[8]	Vancouver, Canada	1996 - 2003	VIDUS	1013	Current unstable housing
Craib et al. (2003)[9]	Vancouver, Canada	1996 - 2000	VIDUS	941	Unstable housing (past 6 months)
Cullen et al. (2015)[10]	England, Wales Northern Ireland, UK	2011	UAM Study	1718	Recent homelessness (past 12 months)
Grebely et al. (2014)[11]	Vancouver, Canada	1996 - 2012	VIDUS	364	Unstable housing (6 months before enrolment)
Hahn et al. (2002)[12]	San Francisco, USA	2000 - 2001	UFO	776	Recent homelessness
Ickowicz et al. (2015)[13]	Vancouver, Canada	1996 - 2015	VIDUS	1927	Recent homelessness (past 6 months)
Jacka et al. (2019)[14]	Montreal, Canada	2004-2017	HEPCO	440	Unstable housing (past 3 months)
Kaberg et al. (2018)[15]	Stockholm, Sweden	2013 - 2016	Stockholm NSP	584	Homelessness (past 6 months)
Kim et al. (2009)[16]	Vancouver, Canada	1996 - 2007	VIDUS	3074	Unstable housing (past 6 months)
Maas et al. (2007)[17]	Vancouver, Canada	1996 - 2004	VIDUS	1587	Unstable housing (past 6 months)
Maher et al. (2006)[18]	New South Wales, Australia	1999 - 2002	Australia NSP	368	Homelessness (past 6 months)
Miller et al. (2006)[19]	Vancouver, Canada	1996 - 2003	VIDUS	1548	Unstable housing (past 6 months)
Miller et al. (2006)[20]	Vancouver, Canada	1996 - 2003	VIDUS	1013	Unstable housing (past 6 months)
Miller et al. (2002)[21]	Vancouver, Canada	1996 -	VIDUS	232	Unstable housing (past 6 months)
Morris et al.[22]	USA, Canada, The Netherlands, Australia	1985 - 2011	Inc3 Collaboration	1391	Unstable housing (past 6 months)
Nelson et al. (2002)[23]	Baltimore, USA	1988 - 1989	ALIVE	1846	Recent homelessness (past 6 months)
Puri et al. (2014)[24]	Vancouver, Canada	2005 - 2011	ARYS	940	Recent homelessness (within 6 months before enrolment)
Puzhko et al. (2017)[25]	Montreal, Canada	2004 - 2011	HEPCO	465	Unstable housing (past 1 month)
Sacks-Davis et al. (2016)[26]	Montreal, Canada	2004 - 2011	HEPCO	1198	Unstable housing (past 3 months)
Scheim et al. (2018)[27]	Vancouver, Canada	1996 - 2014	VIDUS	1131	Recent homelessness (past 6 months)
Shannon et al. (2010)[28]	Vancouver, Canada	1996 - 2007	VIDUS & SEOSI	3074	Unstable housing (past 6 months)
Smith et al. (2015)[29]	San Francisco, USA	1988 - 2008	ALIVE	1904	Recent homelessness (past 6 months)
Spittal et al. (2002)[30]	Vancouver, Canada	1996 - 2000	VIDUS	939	Unstable housing (past 6 months)
Strathdee et al. (2001)[31]	Baltimore, USA	1988 - 1989	ALIVE	1874	Recent homelessness (past 6 months)
Tyndall et al. (2003)[32]	Vancouver, Canada	1996 – 2000	VIDUS	940	Unstable housing (past 6 months)

Van de Berg et al. (2007)[33]	Amsterdam, The Netherlands	1985 - 2005	ACS	1640	Recent homelessness (past 6 months)
Van de Berg et al. (2007)[34]	Amsterdam, The Netherlands	1985 – 2005	ACS	1640	Recent homelessness (past 6 months)
Tsui et al. (2014)[35]	San Francisco, USA	2000 - 2003	UFO study	552	Homelessness (past 3 months)
White et al. (2014)[36]	Sydney, Australia	2008 - 2011	HITS-c	129	Current unstable housing
Wood et al. (2009)[37]	Vancouver, Canada	1996 - 2007	VIDUS & BART	1429	Unstable housing (past 6 months)
Wood et al. (2007)[38]	Vancouver, Canada	1996 - 2004	VIDUS	1035	Unstable housing (past 6 months)
Young et al. (2016)[39]	Vancouver, Canada	1996- 2013	VIDUS	1683	Recent homelessness (past 6 months)

VIDUS= Vancouver Injection Drug Users Study. BART: Barriers to Accessing Antiretroviral Therapy. ALIVE: AIDS Linked to the Intravenous Experience. ARYS= At Risk Youth Study. ACS= Amsterdam Cohort Study. HITS-c= Hepatitis C Incidence and Transmission Study-community.

Appendix table 3: Univariable meta-regression for the effect of recent homelessness or unstable housing on HIV acquisition risk.

	Number of estimates	RR (95% CI)	Ratio (95% CI)	τ^2	Adjusted R^2	P - value
**Region**				0.1391	-18.82	0.5365
Europe	5	1.43 (0.96, 2.15)	1.00			
North America	8	1.53 (1.05, 2.25)	1.11 (0.58, 2.16)			
Asia	3	1.62 (1.19, 2.18)	1.09 (0.47, 2.56)			
East Africa	1	3.45 (1.52, 7.83)	2.51 (0.66, 9.49)			
**Economic level**				0.1218	-4.03	0.383
High income	10	1.43 (1.04, 1.97)	1.00			
Low/Middle income	7	1.79 (1.40, 2.30)	1.26 (0.73, 2.16)			
**Female (%)**				0.1533	64.27%	0.461
Per 10% increase	15		0.91 (0.70, 1.19)			
**Age (mean/median)**				0.1372	-17.17	0.6040
< 33.6 years [a]	7	1.77 (1.37, 2.28)	1.00			
>/= 33.6 years [a]	8	1.54 (1.05, 2.26)	0.88 (0.48, 1.59)			
Not Reported	2	1.14 (0.52, 2.48)	0.67 (0.29, 1.55)			
**Duration of injecting**				0.1353	-15.57	0.6700
< 9.9 years [a]	6	1.83 (1.27, 2.64)	1.00			
>/= 9.9 years [a]	7	1.59 (1.02, 2.49)	0.90 (0.45, 1.77)			
Not Reported	4	1.40 (1.07, 1.84)	0.74 (0.36, 1.54)			
**Baseline HIV prevalence**				0.1471	26.93	0.198
Per 10% increase	12		0.82 (0.60, 1.13)			
**Proportion homeless /unstably housed at baseline**				0.1009	13.85	0.2173
< 25.04% [a]	8	1.30 (0.95, 1.78)	1.00			
>/= 25.04% [a]	8	1.96 (1.42, 2.70)	1.51 (0.92, 2.48)			
Not Reported	1	1.24 (0.71, 2.17)	0.96 (0.36, 2.54)			
**OST coverage at baseline (%)**				0.1375	-17.44	0.5269
< 11.92% [a]	5	1.79 (1.39, 2.31)	1.00			
>/= 11.92% [a]	5	1.39 (0.90, 2.15)	0.71 (0.35, 1.42)			
Not Reported	7	1.41 (0.91, 2.16)	0.74 (0.38, 1.43)			
**Study design**				0.1195	-2.04	0.316
Longitudinal	15	1.62 (1.27, 2.07)	1.00			
Cross-sectional	2	1.10 (0.51, 2.38)	0.69 (0.32, 1.49)			
**Effect type**				0.1257	-7.37	0.4267
Hazard ratio	10	1.56 (1.08, 2.25)	1.00			
Incidence rate ratio	5	1.70 (1.38, 2.10)	1.14 (0.64, 2.04)			
Odds ratio	1	1.24 (0.71, 2.17)	0.79 (0.28, 2.23)			
Risk ratio	1	0.70 (0.33, 1.52)	0.45 (0.14, 1.47)			
**Start of study**				0.1289	-10.09	0.701
Before 2005 [a]	7	1.47 (0.97, 2.21)	1.00			
On/After 2005 [a]	10	1.67 (1.35, 2.10)	1.10 (0.66, 1.84)			
**Midpoint of study**				0.1275	-8.87	0.443
Before 2008 [a]	7	1.37 (0.86, 2.17)	1.00			
On/After 2008 [a]	10	1.68 (1.41, 2.00)	1.21 (0.72, 2.02)			
**Duration of study**				0.1325	-13.19	0.883
< 4 years [a]	8	1.57 (1.18, 2.08)	1.00			
>/= 4 years [a]	9	1.58 (1.11, 2.24)	1.04 (0.61, 1.77)			

**Definition of homelessness (time frame of assessment)**				0.1135	3.06	0.2583
Past 6 months	9	1.57 (1.07, 2.31)	1.00			
Current	6	1.70 (1.40, 2.07)	1.09 (0.64, 1.84)			
Past 12 months	2	0.75 (0.38, 1.48)	0.49 (0.18, 1.30)			
**Publication status**				0.1220	-4.25	0.631
Unpublished	12	1.46 (1.12, 1.91)	1.00			
Published	5	1.65 (1.11, 2.44)	1.12 (0.67, 1.89)			
**Ever incarcerated (%)**				0.1487	-26.98	0.9653
< 71.3% [a]	4	1.70 (1.31, 2.20)	1.00			
>/= 71.3% [a]	4	1.63 (0.79, 3.39)	0.93 (0.40, 2.15)			
Not Reported	9	1.53 (1.13, 2.08)	0.91 (0.44, 1.89)			
**Recently incarcerated (%)**				0.1325	-13.15	0.8617
< 12.0% [a]	3	1.74 (1.19, 2.54)	1.00			
>/= 12.0% [a]	3	1.61 (0.50, 5.19)	0.79 (0.31, 1.99)			
Not Reported	11	1.59 (1.24, 2.03)	0.90 (0.44, 1.82)			

a: median values

Appendix table 4: Univariable meta-regression for the effect of recent homelessness or unstable housing on HCV acquisition risk.

	Number of studies	RR (95% CI)	Ratio (95% CI)	τ^2	Adjusted R^2	P -value
**Region**				0.03329	40.77	0.0261
Europe	13	2.06 (1.64, 2.59)	1.00			
North America	10	1.59 (1.34, 1.88)	0.77 (0.58, 1.03)			
Australasia	4	1.27 (0.81, 1.97)	0.62 (0.36, 1.08)			
South and Central Asia	1	0.76 (0.45, 1.29)	0.37 (0.18, 0.75)			
**Economic level**				0.03721	33.79	0.092
High income	26	1.71 (1.44, 1.90)	1.00			
Low/Middle income	2	1.10 (0.48, 2.55)	0.60 (0.33, 1.09)			
**Female (%)**				0.06981	-14.34	0.739
Per 10% increase	25		1.03 (0.86, 1.23)			
**Age (mean/median)**				0.06547	-16.49	0.3276
< 27.95 years [a]	11	1.49 (1.24, 1.78)	1.00			
>= 27.95 years [a]	10	1.86 (1.24, 2.78)	1.30 (0.91, 1.86)			
Not Reported	7	1.83 (1.51, 2.22)	1.21 (0.80, 1.84)			
**Duration of injecting (mean/median)**				0.0558	0.71	0.3461
< 6.7 years [a]	7	1.38 (1.01, 1.87)	1.00			
>= 6.7 years [a]	7	1.87 (1.43, 2.45)	1.35 (0.87, 2.10)			
Not Reported	14	1.71 (1.40, 2.07)	1.23 (0.85, 1.79)			
**Baseline HCV prevalence**				0.1491	-9.40	0.549
Per 10% increase	18		1.04 (0.91, 1.18)			
**Proportion homeless/unstably housed at baseline**	13	1.69 (1.26, 2.26)	1.00	0.06636	-18.08	0.8940
< 27.2% [a]	13	1.65 (1.42, 1.92)	0.95 (0.67, 1.34)			
>/= 27.2% [a]	2	1.70 (0.65, 4.48)	0.86 (0.42, 1.75)			
Not Reported						
**OST coverage at baseline**				0.03559	36.67	0.0136
< 36.5% [a]	8	1.78 (1.53, 2.10)	1.00			
>/= 36.5% [a]	8	2.68 (1.88, 3.81)	1.53 (1.00, 2.37)			
Not Reported	12	1.39 (1.14, 1.69)	0.81 (0.61, 1.09)			
**Study design**				0.06382	-13.55	0.315
Longitudinal	25	1.61 (1.40, 1.86)	1.00			
Cross-sectional	3	2.12 (1.05, 4.30)	1.27 (0.79, 2.04)			
**Effect type**				0.06993	-24.43	0.5913
Hazard ratio	18	1.59 (1.36, 1.86)	1.00			
Incidence rate ratio	6	1.91 (1.38, 2.65)	1.25 (0.78, 2.01)			
Risk ratio	4	1.79 (1.03, 3.11)	1.12 (0.72, 1.76)			
**Start of study**				0.06422	-46.77	0.498
Before 2004 [a]	13	1.59 (1.34, 1.89)	1.00			
On/After 2004 [a]	15	1.74 (1.39, 2.18)	1.11 (0.81, 1.52)			
**Midpoint of study**				0.06091	-8.37	0.579
Before Mid-2007 [a]	14	1.57 (1.28, 1.93)	1.00			
On/After Mid-2007 [a]	14	1.71 (1.40, 2.08)	1.09 (0.79, 1.50)			

**Length of study**				0.02262	59.76	0.035
< 4.5 years [a]	13	1.36 (1.06, 1.75)	1.00			
>/= 4.5 years [a]	15	1.83 (1.59, 2.11)	1.37 (1.02, 1.84)			
**Definition of homelessness**				0.05409	3.76	0.4628
Past 6 months	12	1.48 (1.18, 1.86)	1.00			
Current	7	1.47 (1.04, 2.08)	1.00 (0.61, 1.64)			
Past 12 months	5	1.63 (1.24, 2.15)	1.14 (0.74, 1.77)			
Past 3 months	3	2.05 (1.68, 2.50)	1.37 (0.86, 2.17)			
Past 1 month	1	2.34 (1.72, 3.18)	1.57 (0.82, 2.98)			
**Publication status**				0.06370	-13.33	0.886
Unpublished	17	1.69 (1.49, 1.92)	1.00			
Published	11	1.61 (1.18, 2.19)	0.98 (0.70, 1.36)			
**Ever incarcerated**				0.06853	-21.94	0.7954
**(%)**	7	1.46 (1.16, 1.84)	1.00			
< 60.1% [a]	7	1.79 (1.26, 2.54)	1.17 (0.73, 1.88)			
>/= 60.1% [a]	13	1.66 (1.36, 2.02)	1.10 (0.72, 1.68)			
Not Reported						
**Recently incarcerated (%)**				0.07168	-27.53	0.8522
< 19.5% [a]	4	1.55 (1.20, 2.01)	1.00			
>/= 19.5% [a]	5	1.75 (1.40, 2.30)	1.18 (0.66, 2.05)			
Not Reported	19	1.65 (1.36, 2.00)	1.12 (0.68, 1.83)			

a: median values

Appendix table 9: Analysis of confounding: univariable meta-regression for the adjusted effect of recent homelessness or unstable housing on the risk of HIV acquisition.

	Number of studies	RR (95% CI)	Ratio (95% CI)	τ^2	Adjusted R^2	P - value
**Adjusted for recent OST exposure**				0.05845	43.27	0.129
No	2	1.91 (1.47, 2.48)	1.00			
Yes	7	1.22 (0.91, 1.63)	0.64 (0.35, 1.18)			
**Adjusted for recent incarceration history**				0.02838	72.45	0.034
No	3	1.92 (1.51, 2.46)	1.00			
Yes	6	1.13 (0.85, 1.51)	0.58 (0.36, 0.95)			
**Adjusted for stimulant injecting**				0.101	1.99	0.342
No	2	1.78 (1.25, 2.53)	1.00			
Yes	7	1.30 (0.93, 1.81)	0.71 (0.32, 1.57)			

Appendix table 10: Analysis of confounding: univariable meta-regression for the adjusted effect of recent homelessness or unstable housing on the risk of HCV acquisition.

	Number of studies	RR (95% CI)	Ratio (95% CI)	τ^2	Adjusted R^2	P - value
**Adjusted for recent OST exposure**				0.0132	-235.08	0.421
No	3	1.33 (0.80, 2.22)	1.00			
Yes	11	1.67 (1.45, 1.92)	1.17 (0.77, 1.76)			
**Adjusted for recent incarceration history**				0.007044	-78.86	0.764
No	8	1.72 (1.23, 2.41)	1.00			
Yes	6	1.60 (1.43, 1.89)	1.05 (0.74, 1.49)			
**Adjusted for recent stimulant injecting**				0.007221	-83.36	0.398
No	4	1.81 (1.41, 2.32)	1.00			
Yes	9	1.59 (1.33, 1.90)	0.87 (0.61, 1.24)			

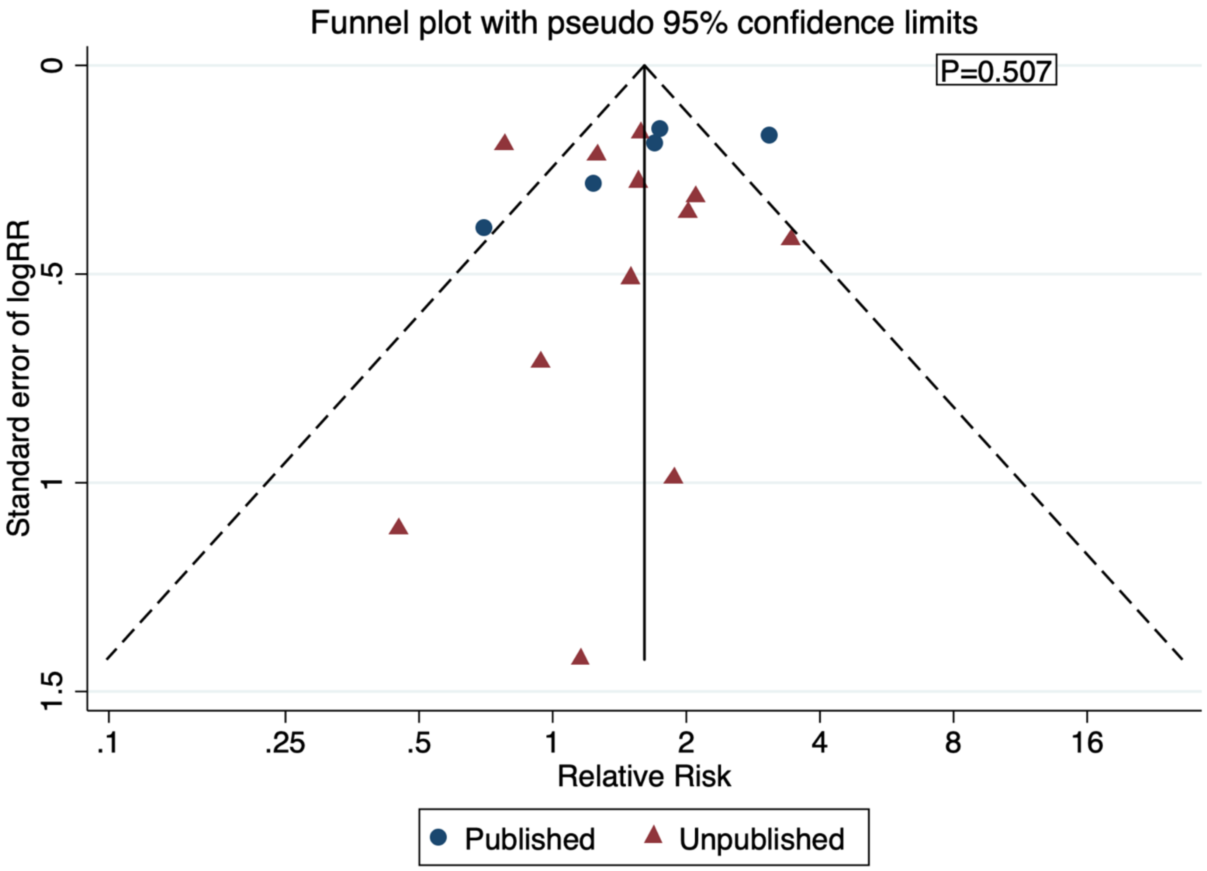

Appendix Figure 1: Funnel plot of studies included in the meta-analysis of the effect of recent homelessness and unstable housing on HIV acquisition risk with box showing p-value for the Egger's test for funnel plot asymmetry.

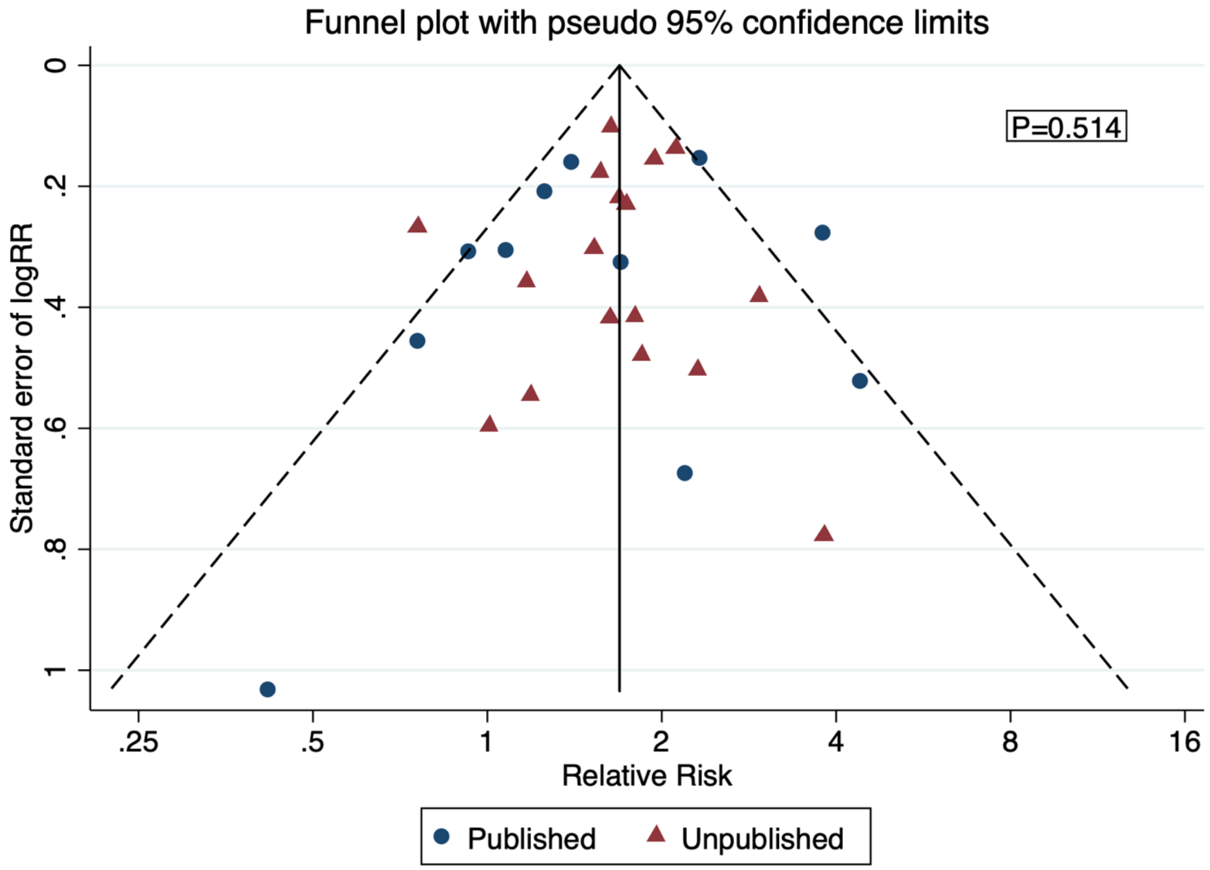

Appendix Figure 2: Funnel plot of studies included in the meta-analysis of the effect of recent homelessness and unstable housing on HCV acquisition risk with box showing p-value for the Egger's test for funnel plot asymmetry.

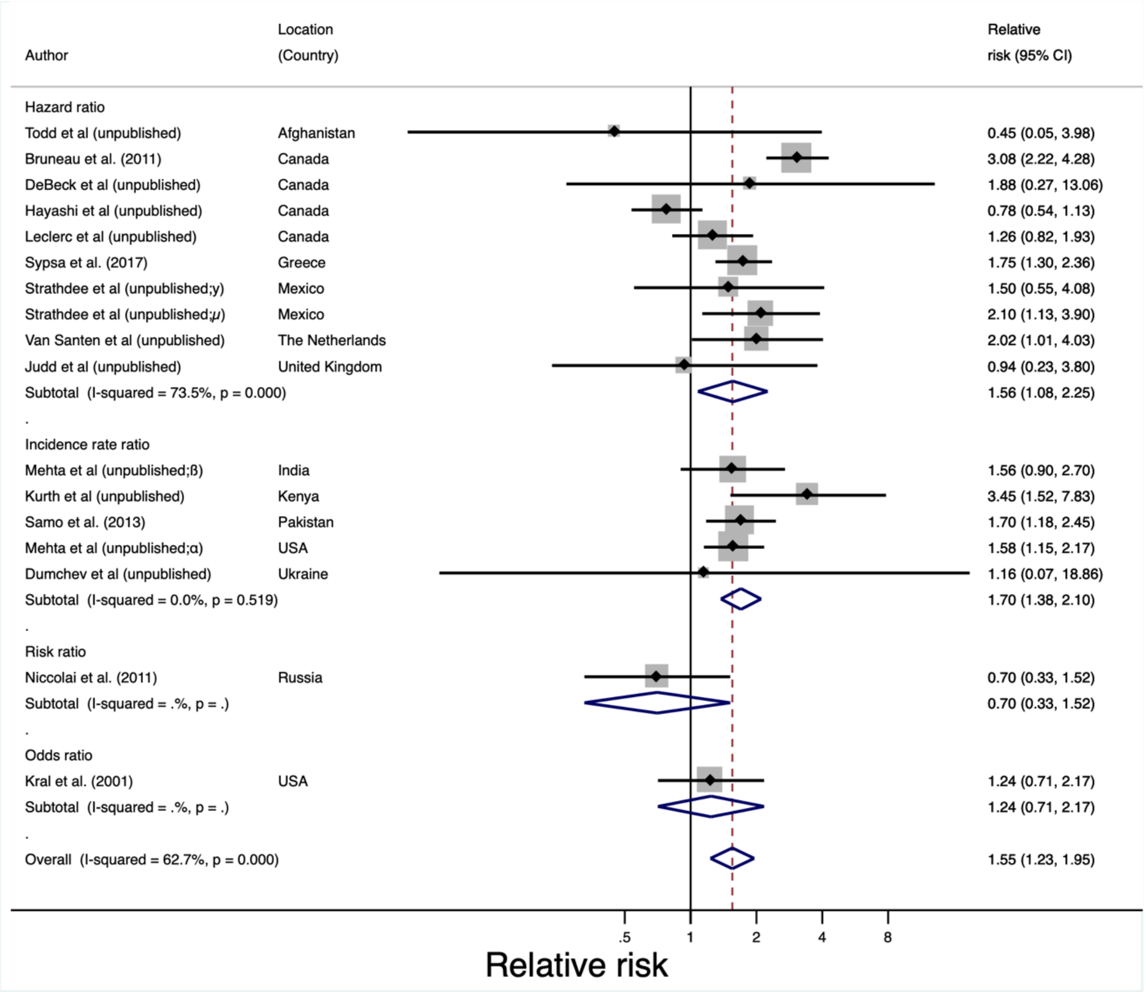

Appendix figure 3: Meta-analysis of studies showing crude effect of recent homelessness or unstable housing on risk of HIV acquisition among PWID, by effect type.

α=AIDS Linked to the Intravenous Experience (ALIVE). ß=Indian community survey (Indian ICC). y= El Cuete III (EC3). µ= El Cuete IV (EC4).

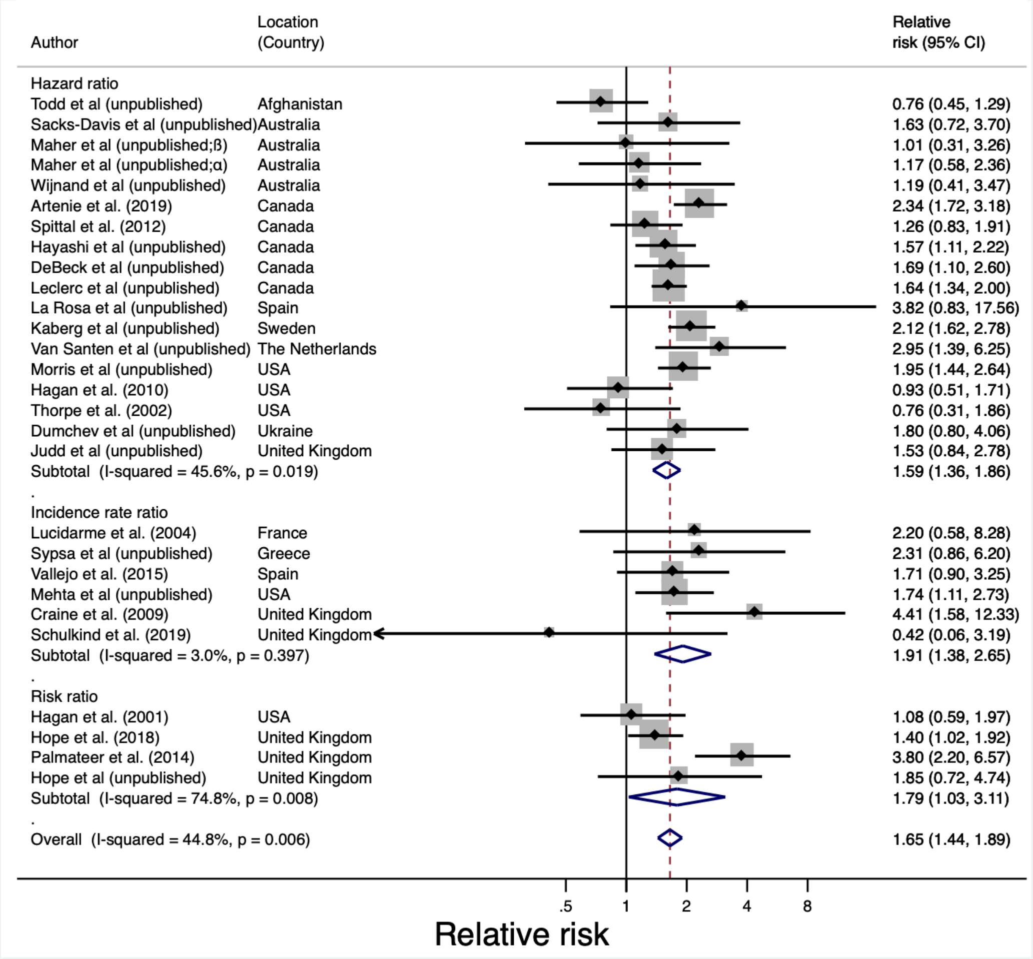

Appendix figure 4: Meta-analysis of studies showing crude effect of recent homelessness or unstable housing on HCV acquisition risk among PWID, by effect type.
α=Hepatitis C Incidence and Transmission Study-community (HITS-c). ß= Hepatitis C Virus Cohort (HCVC).

Appendix table 5: Characteristics of included studies for the effect of recent homelessness or unstable housing on HCV acquisition risk

Author/Publication Year	Cohort name, recruitment sites and methods, inclusion and exclusion criteria	Definition of homelessness or unstable housing (time frame of assessment)	Effect estimates
Artenie et al. (2019)[40]	**HEPCO** includes HCV- and HIV-negative participants already followed up in the St Luc cohort (30%), as well as new participants recruited through street-level strategies such as word of mouth (36%) or through community program referrals (34%). To be eligible, participants must report having injected drugs within the previous 6 months, living in the Greater Montréal area [Bruneau Addiction 2018] and be 18 years of age or older. Initially, only HCV-seronegative participants, at risk of primary HCV infection, were recruited. Since 2011, recruitment expanded to include HCV-seropositive, RNA-negative people who inject drugs, who had cleared their infection and were at risk of re-infection. Eligibility for the present study was restricted to HEPCO participants who reported using opioids or taking opioid agonist treatment at least at 1 study visit, and who had a minimum of 2 total visits.	Unstable housing in the past month	HR 2.34 (1.72-3.17)<br>aHR 2.14 (1.54-2.96)<br>Adjusted for: Dosage of OAT and perceived adequacy, sex, duration of injection drug use, cocaine injection, incarceration history, previous HCV infection.
Craine et al. (2009)[41]	IDUs were recruited from a range of field stations across South Wales. These included treatment ser- vices, needle and syringe exchange services and home- less hostels. Individuals were also approached on the street. Drug injectors were invited to enter the study by professional staff, by researchers and by word of mouth between study participants. Recruitment was thus opportunistic but made use of existing social and drug-using networks. The target criteria for inclusion in the study were being a current or a recent drug injector.	Homelessness in the past 12 months	IRR 4.41 (1.60-12.5)<br>aIRR 2.9 (1.02-8.28)<br>Adjusted for: In OST at follow-up, any equipment haring in the past year, sharing needles and syringes in past year, population size of region (<200,000 vs >200,000)
Debeck et al (unpublished)[24]	**ARYS** participants were recruited through snowball sampling and extensive street-based outreach methods. The street-based recruitment approach produced a sample of youth who spent extensive time on the streets (a large proportion of whom were homeless) of Vancouver and from youth agencies and services. To be eligible, participants at recruitment must have been aged 14-26 years; used illicit drugs other than marijuana in the past 30 days; be "street involved," defined as having been homeless in the past 6 months or recently having used a service for street-involved youth (e.g., housing or nutrition support); and had to provide written informed consent; and had to provide written informed consent. For this analysis, participants were eligible as of the first visit they reported injection in the previous 6 months and if they were seronegative for anti-HCV.	Homelessness in the past 6 months	HR 1.69 (1.1-2.6)<br>aHR 1.45 (0.92-2.28)<br>Adjusted for: Recent incarceration, MMT coverage, crack injecting.
Dumchev et al (unpublished)	**Ukraine cohort,** Clients were recruited using respondent driven sampling. Initial seeds (5 per site) were recruited at harm reduction programmes. Participants of the prospective cohort had to meet the following criteria: (1) being HIV negative at the time of screening, confirmed by HIV rapid testing; (2) age of 16 or older by self-report; (3) have visible signs of recent injection, verified by the study nurse; (4) ability to provide informed consent; (5) willingness to participate in the study for 18 months and provide contact information; (6) intention to live in the city of recruitment for 18 months; (7) Presence of a client record in SYREX.	Current homelessness	HR 1.80 (0.80-4.07)<br>aHR 1.57 (0.69-3.54)<br>Adjusted for: Ever prison, age (continuous), IDU duration (continuous)
Hayashi et al (unpublished)[27]	**VIDUS,** The Vancouver Injection Drug Users Study (VIDUS) is an open prospective community-recruited cohort of PWID in Vancouver, Canada. Beginning in May 1996, active PWID (i.e. those who reported injecting drugs in the previous month) were recruited in the Greater Vancouver region on an ongoing basis throughout the study period. Recruitment strategies employ extensive street-based outreach and ''snowball'' sampling approaches. Given VIDUS is an open cohort, new participants were continuously enrolled in the cohort over the study period to replace those who died or were lost to follow-up. All participants were recruited through street outreach, word of mouth, and self-referral, and provided written informed consent prior to entering the study. Participants were eligible if they had injected illicit drugs at least once in the previous month, resided in the greater Vancouver region, and provided written informed consent. For this analysis, participants were eligible if they were seronegative for anti-HCV.	Homelessness in the past 6 months	HR 1.57 (1.11-2.22)<br>aHR 1.62 (1.14-2.29)<br>Adjusted for: Recent incarceration, MMT coverage, crack injecting

Hagan et al. (2001)[42]	**RAVEN Study,** beginning in June 1994, cohort study subjects were recruited from six drug treatment programs and from social service, corrections, and drug-use assessment agencies. In each setting, subjects were systematically selected by use of a random-number based scheme from 1) all agency clients present during recruitment hours (non-drug treatment settings), or 2) all newly enrolled drug treatment clients. Series of random numbers between one and nine were issued to interviewers who would select the nth client as he or she entered the agency or appeared on client lists. Eligibility criteria included having injected an illicit drug in the previous year, being English or Spanish speaking, being 14 years or older, and not being already enrolled in the study.	Homelessness in the past 12 months	RR 1.08 (0.59-1.97)
Hagan et al. (2010)[43]	**Drug User Intervention Trial (DUIT),** Recruitment for this study took place between May 2002 and January 2004 in Baltimore, Seattle, Los Angeles, New York and Chicago. Street outreach, advertising, and coupon-based participant referrals were used to recruit young active injectors. To be eligible, individuals were required to have injected an illicit drug in the past 6 months, reside in the recruitment city with no plans to move within 12 months, and be English-speaking, aged 15–30 years, and seronegative for HIV and HCV antibody. A baseline screening visit included a behavioural assessment interview followed by HIV and HCV antibody testing. Individuals who tested negative for both infections were invited to enrol in the trial. This analysis of HCV seroconversion included eligible subjects who enrolled in the trial and completed follow-up study visits within 12 months of the baseline. Data analysis was also restricted to subjects who reported injecting during the follow-up period, because we were not interested in examining HCV seroconversion associated with behaviour unrelated to drug injection.	Homelessness in the past 6 months	HR 0.93 (0.68-2.29)
Hope et al. (2018)[44]	**UAM Study,** PWID across England, Wales and Northern Ireland are recruited into an annual cross-sectional, unlinked anonymous bio-behavioural survey (the UAM Survey); people who have ever injected drugs are recruited through specialist services for PWID providing advice, NSPs, OST or addiction treatment. Service selection reflects the range of services provided for PWID and what is known about geographic variations in drug use. Those agreeing to participate self-complete a short questionnaire and provide a dried-blood spot (DBS) sample at the collaborating service. DBS collection involves obtaining a few drops of blood, through a lancet prick to the finger, onto absorbent filter paper (PerkinElmer 226). In this study, we included only individuals recruited between 2011 and 2013 inclusive who had injected during the year preceding survey participation. Samples that were anti-HIV positive (n = 25) were excluded, as the effects of HIV on the immune system is likely to affect anti-HCV avidity.	Homelessness in the past 12 months	RR 1.40 (1.02, 1.92)
Hope et al (unpublished)[45]	**UK Community Surveys** recruited IDUs through respondent-driven sampling (RDS). Eligibility criterion was individuals who had injected drugs in the last 4 weeks.	Homelessness in the past 12 months	RR 1.85 (0.72-4.73)<br>aRR 1.62 (0.55-4.56)<br>Adjusted for: Recent incarceration (last 12 months), current OST status, Use of cocaine, duration of injecting (<3 years, 3-5 years, 5-10 years, 11+ years).
Judd et al (unpublished)[46]	**London Cohort,** Recruitment was done in the community settings. In 2001, we recruited from community settings mainly in London, but also in Brighton, 428 injecting drug users who were aged below 30 years or had been injecting for six years or fewer. All had injected in the previous four weeks and could provide addresses for follow up.	Unstable housing in the past 12 months	HR 1.53(0.84-2.77)
Kaberg et al (unpublished)[47]	**Stockholm NEP,** Participants were recruited from the Stockholm Needle Exchange Program. All participants attending the NEP were included.	Homelessness in the past 3 months	HR 2.12 (1.62-2.78)
La Rosa et al (unpublished)[48]	**Mobile Harm Reduction Unit, Madrid,** The MHRU attends PWUD actively street outreach who have limited access to standard healthcare. Only individuals who were HCV-negative at baseline, and who had at least one follow-up visit (to re-test for HCV infection) were eligible for the analysis of HCV incidence density. Additionally, individuals were included if they had a history of injecting.	Current homelessness	HR 3.82 (-.80-16.9)<br>aHR 4.90 (1.07-23.1)<br>Adjusted for: Crack injecting, OST exposure
Leclerc et al (unpublished)[49]	**SurvUDI,** Participants are recruited in urban areas, including Montréal and neighbouring South Shore, Québec City, the Hull-Ottawa region, and 5 semi-urban areas of the province of Québec. Overall, since 2004, 94.6% of participants were recruited in harm reduction programs. Others were recruited in drop-in centres, detention centres, detoxification clinics, and rehabilitation programmes. Eligibility criteria include being aged 14 years or older, injecting at least once within the past 6 months, speaking French or English and being able to provide informed consent.	Unstable housing in the past 6 months	HR 1.64 (1.34-2.00)<br>aHR 1.44 (1.16-1.78)<br>Adjusted for: Living in jail in past 6 months, OST exposure, syringe sharing

Lucidarme et al. (2004)[50]	***France Cohort,*** the participants recruited were drug-user attendees of six care centres in Northern and Eastern France. Eligible participants were those who had injected drugs at least once in their lifetime and whose HCV serology was presumed to be negative. Sixty-three persons positive for anti-HCV and 32 whose serological status was unknown were excluded from follow-up. Of the 231 HCV antibody negative IDUs enrolled in the study, three (2%) died and 63 (27%) did not undergo a final serum test and were excluded from the analysis.	Unstable housing in the past 3 months	IRR 2.20 (0.51-7.22)
Maher et al (unpublished)[51]	**HCVC,** Participants were recruited using a variety of strategies (direct approaches, word-of-mouth and fliers) designed to identify a broad cross-section of potential participants across a range of settings, including street-based outreach, local methadone and sexual health clinics and NSPs. Eligibility criteria include injected drugs in the last 6 months and anti-HCV serostatus not known to be positive. Individuals that tested antibody HCV negative were enrolled in the study.	Unstable housing in the past 6 months	HR 1.01 (0.31-3.23)
Maher et al (unpublished)[52]	**Hepatitis C Incidence and Transmission Study-community [HITS-c],** Snowball sampling techniques based on social and drug-use networks, including incentives for peer referral and targeted outreach sampling. Eligibility criteria for study screening were age 16 years or older; self-reported HCV antibody-negative or unknown status; injection of drugs in the past 12 months; and willingness to provide contact details. PWID satisfying all inclusion criteria (anti-HCV antibody, hepatitis B surface antigen and anti- HIV antibody tests all negative) were considered eligible for HITS-c enrolment for 6 weeks after the screening visit.	Unstable housing in the past 6 months	HR 1.17 (0.58-2.36)
Mehta et al (unpublished)[29]	**ALIVE,** Participants were recruited into the study from various agencies that served intravenous drug users. These agencies include Maryland Division of Parole and Probation, the Baltimore City Health Department's STD clinics, hospital emergency rooms, and homeless shelters in Baltimore. To extend recruitment beyond these agencies, outreach efforts were developed in collaboration with the Street Outreach AIDS Prevention (SOAP) Unit of the Health Education Resource Organization (HERO), a community AIDS education group in Maryland. The SOAP unit include ten recovered and recovering addicts who provide AIDS education to the inner-city community through contacts on the street. These workers distributed brochures and answered questions about the ALIVE study. Study staff also distributed brochures at local public housing projects and other public places known to be frequented by IVDUs. In summary, recruitment sources include: Word-of-mouth, drug treatment programs, HERO Street Outreach, Parole and Probation, STD Clinics, HIV clinics, and emergency rooms. Between 1998-1999, all participants acknowledged non- medical injection-drug use within the preceding 11 years, were >18 years of age, and were free of AIDS at entry into the study. additional persons were recruited into this cohort in 1994–1995 (n= 391), 1998 (n= 244), and 2005–2008 (n= 875). Some recruitment criteria changed over time. In the fourth period, persons were no longer required to be AIDS-free at entry. To replenish with active injectors, in 1994–1995, persons had to have injected in the preceding 3 years, and in 1998 and 2005–2008, in the preceding year.	Homelessness in the past 6 months	IRR 1.74 (1.11-2.73)<br>aIRR 1.66 (1.01-2.74)<br>Adjusted for: Cocaine, Jail, OST/MAT treatment
Morris et al (unpublished)[53]	**UFO,** Recruitment was done by outreach and by word of mouth. Cohort eligibility was restricted to those <30 years old, those who reported injecting drugs in the prior month, those who spoke English as their primary language, and those who, if recruited in 2003 or later, did not plan to travel outside of San Francisco within the next 3 months.	Homelessness in the past 3 months	HR 1.95 (1.44-2.64)<br>aHR 1.65 (1.21-2.25)<br>Adjusted for: Gender, age, injecting frequency, recent unsafe injecting behaviours, and number of injecting partners
Palmateer et al. (2014)[54]	**NESI,** The Needle Exchange Surveillance Initiative (NESI) is a voluntary anonymous cross-sectional survey of PWID undertaken across mainland Scotland. Between June 2008 and June 2009, participants were recruited from 22 agencies and 81 pharmacies that provide sterile injecting equipment (these sites may also provide other harm reduction interventions, such as OST), which comprise 42% of IEP services in Scotland (ISD Scotland, 2010). Within logistical constraints (service manager agreement and a private room where the interviews could take place), services were selected to be broadly geographically representative. Eligible individuals had injected drugs in the past and had not participated in the study during the current survey year. Current injectors (defined as having injected in the last 6 months) were oversampled, if necessary, so that the proportion of the sample comprised by this group was at least 75% in each recruitment area. People who had ever injected drugs were eligible to participate.	Homelessness in the past 6 months	RR 3.80 (2.20-6.57)
Sacks-Davis et al (unpublished)[55]	**Network 2,** Between 2005 and 2006, PWID who had injected in the previous six months were recruited from major street drug markets located across metropolitan Melbourne using modified snowball sampling**.**	Current unstable housing	HR 1.63 (0.72-3.70)<br>aHR 1.58 (0.66-3.79)

	inclusion criteria were aged ≤25 years, duration of injecting less than four years, tested negative for HCV antibodies (anti-HCV), tested HCV RNA negative.		Adjusted for: OST (any pharmacotherapy in the past 3 months), type of infection (primary, reinfection), correlation within individuals.
Schulkind et al. (2019)[56]	**Eradicate,** a prospective observational study conducted at the largest Needle and Syringe Program (NSP) in Dundee, Scotland. Inclusion criteria: age 18-70 y, active HCV positive infection confirmed with PCR, current injecting drug use established through review of needle injection sites and patient history; if female, negative urine test for pregnancy and on Long-Acting reversible contraceptive during study. Exclusion criteria: aggressive or violent behaviour, features of decompensated liver failure, evidence of primary hepatocellular carcinoma, pregnancy, breastfeeding or pre-menopausal female not using effective contraception, contraindication to peg-interferon and ribavirin, previous treatment with peg-interferon and ribavirin, participation in a drug study within previous 30 days, and inability to provide informed consent.	Current Unstable Housing	IRR: 0.42 (0.056-3.23)
Spittal et al. (2012)[57]	**The CEDAR Project**, Participants living primarily in the downtown areas of both cities of Vancouver and Prince George, British Columbia were recruited through referral by health care providers, community outreach and word of mouth. Eligibility criteria for study entry included age 14 to 30 years and to have smoked or injected illicit drugs, aside from marijuana, in the month prior to enrolment. The Cedar Project cohort includes 605 participants in total, however this study included only participants who reported injection drug use, were HCV negative at baseline, reported injection drug use and who returned for at least one of eight follow-up interviews up to December 2008 (n = 148).	Homelessness in the past 6 months	HR 1.26 (0.83-1.90)
Sypsa et al (unpublished)[58]	**ARISTOTLE HCV-HIV,** Participants were recruited through respondent-driven sampling. A dual incentive system was used in which participants received incentives for participating in the programme (primary incentives) as well as for recruiting others (secondary incentives). Seeds were recruited by staff of the Greek Organization Against Drugs (OKANA) who had experience in working with the target population. The programme itself was implemented in a building of the OKANA, located in the centre of Athens. The staff included a physician, inter-viewers with prior experience with the target population and a psychologist and two social workers. A flow manager was responsible for maintaining the flow of participants and ensuring that participants completed each of the steps of the process on a first-come, first-served basis. Five RDS rounds were implemented. The selection of seeds was based on whether the candidates were well connected to other members of the target population, liked by their peers and with motivation to the programme. The aim was to enrol seeds diverse in terms of country of origin, gender and HIV status to ensure reaching equilibrium. Respondents were eligible to participate if they presented a valid RDS coupon, had injected drugs without a prescription in the past 12 months, were 18 years of age and over, and resided in the Athens metropolitan area. A subset of PWID participated in multiple RDS rounds, allowing for assessment of HCV seroconversion. Only individuals with ≤2 years injecting were tested for anti-HCV.	Current homelessness	IRR 2.31 (0.86-6.19)
Thorpe et al. (2002)[59]	**CIDUS-Chicago** study was conducted from storefront offices in four low-income Chicago neighbourhoods, each selected for its high concentration of drug users and its distinct racial and ethnic composition. Participants were recruited through street outreach, targeted advertising, and peer referrals. Street recruiting by former IDUs was done in areas such as youth hangouts, "shooting galleries" (places where drug users gather to inject drugs and perhaps be assisted in injecting), and illicit drug markets. Advertisements were placed in alternative magazines, in newspapers, and on college campuses. In a version of respondent-driven sampling, each newly interviewed participant received three coupons to distribute to eligible peers. When a peer redeemed one of these numbered coupons by enrolling in the study, the peer recruiter received an incentive fee of $10. Persons were enrolled in the study if they had proof of an eligible birthdate and reported having injected drugs in the past 6 months. Recent injection drug use was verified by inspecting for stigmata, such as scars or abscesses. When stigmata were absent, we interviewed enrolees to ascertain their familiarity with injection routines. This analysis included all participants who were susceptible to hepatitis C infection (negative for antibodies to HCV) at the initial visit.	Homelessness in the past 6 months	HR 0.76 (0.31-1.86)<br>aHR 0.63 (0.25-1.58)<br>Adjusted for: Injection related risk exposures (sharing cookers, sharing cotton filters, sharing rinse water, sharing syringes), demographic covariates (high school diploma, suburban residence), drug use covariates (daily injection in the past 6 months, cocaine injection in the past 6 months).

Todd et al (unpublished)[60]	**Kabul Harm reduction program,** Participants were recruited consecutively from areas of known drug user congregation and through harm reduction programs on scheduled days loosely apportioned by number of IDUs present at those locations, in a variant of time-location sampled. Recruitment sites were initially determined through formative work and through harm reduction programs, with locations adjusted monthly based on information from the participants, the harm reduction field workers, and pharmacists of new areas of congregation. Sites were distributed throughout Kabul city, with most sites in the western section of the city, consistent with reported and confirmed IDU presence throughout the study period. Eligibility was limited to IDUs who were aged ≥18 years; reported injecting drugs within the prior 30 days; residing in Kabul; Dari or Pashto speakers; and able to provide informed consent.	Homelessness in the past 6 months	HR 0.76 (0.45-1.29)
Vallejo et al. (2015)[61]	**Heroin-Itinere cohort,** Street recruitment used targeted sampling and chain-referral methods. Each city was ethnographically mapped, providing a systematic frame with a wide range of street drug scenes. Anthropologists and social workers recruited initial seeds of nominators and participants. Incentive-driven procedures were used. Eligibility: 30 years or younger, use of heroin at least 12 days in the past 12 months and at least 1 day in the past 3 months.	Unstable housing in the past 12 months	IRR 1.71 (0.90-3.25)
Van Santen et al (unpublished)[62]	**Amsterdam Cohort Study**, Participants were recruited mainly through low-threshold methadone programs (including a weekly sexually transmitted disease clinic for drug-using sex workers) and by word of mouth. Persons with either past or current use of illicit drugs (heroin, cocaine, and/or amphetamines) were eligible for recruitment. Since January 2001, only young participants (≤30 years of age) enter the study, but data collection continued for those enrolled before that date.	Homelessness in the past 6 months	HR 2.95 (1.39-6.23)<br>aHR 3.04 (1.42-6.52)<br>Adjusted for: methadone dosing [no methadone vs <60mg vs >=60mg; time updated]
Wijnand et al (unpublished)[63]	**SuperMix,** Participants were recruited in urban Melbourne through respondent-driven sampling (RDS), street outreach and snowball sampling. Eligibility criteria included reporting regular heroin or methamphetamine injection in the past 6 months, being aged >18 years and providing a valid Medicare (Australia's universal healthcare system) number and contact details for data linkage. Two further eligibility criteria that aimed to recruit participants who were young (aged <31 years) and not prescribed OST were withdrawn during early recruitment owing to the ageing PWID population in Melbourne and fluctuating drug-market conditions.	Current homelessness and unstable housing	For homelessness - HR 1.18 (0.16-8.86)<br>aHR 1.09 (0.14-8.59)<br>For unstable housing-HR 1.19 (0.41-3.48)<br>aHR 1.21 (0.40-3.68)<br>Adjusted for: Ice/crystal/shabu injected in the past month, OST treatment.

Appendix table 6: Characteristics of included studies for the effect of recent homelessness or unstable housing on HIV acquisition risk

Author/Publication Year	Cohort name, recruitment sites and methods, inclusion and exclusion criteria	Definition of homelessness or unstable housing (time frame of assessment)	Effect estimates
Bruneau et al. (2011)[64]	**St. Luc Cohort,** First- and second-wave cohort participants volunteered to participate in response to direct street-level recruitment or word-of-mouth referral (57%) or community programs (43%)." Recruitment criteria for the St. Luc Cohort included being 18 years of age or older and having injected drugs within the past 6 months. First wave participants recruited prior to 1992 were excluded because of changes in the questionnaire used since 1992.	Unstable housing in the past 6 months	HR 3.08 (2.22-4.28)<br>aHR 2.07 (1.47-2.90)<br>Adjusted for: age >=30 years (No vs Yes), gender (male vs female), cocaine use in the past month(No vs Yes), heroin use in the past month(No vs Yes), sharing syringes with a person known to be HIV positive (No vs Yes), "booting" (No vs Yes), having sex with a person known to be HIV positive (No vs Yes), period of recruitment (1992-2001 vs 2004 - 2008), NEP participation (No vs Yes), Obtaining 100% syringes from a safe source: recruited during 1992-2001;recruited during 2004-2008)
Debeck et al (unpublished)[24]	**ARYS** participants were recruited through snowball sampling and extensive street-based outreach methods. This includes extensive street-based outreach including outreach during the night-time, and efforts to have street youth recruit their peers. Outreach has also been systematically undertaken in a range of neighbourhoods around the city where street youth are known to congregate. The street-based recruitment approach produced a sample of youth who spent extensive time on the streets (a large proportion of whom were homeless) of Vancouver and from youth agencies and services. To be eligible, participants at recruitment must have been aged 14-26 years; used illicit drugs other than marijuana in the past 30 days; be "street involved," defined as having been homeless in the past 6 months or recently having used a service for street-involved youth (e.g., housing or nutrition support); and had to provide written informed consent; and had to provide written informed consent. For this analysis, the first record where a participant reported injection in the L6M was considered baseline.	Homelessness in the past 6 months	HR 1.88 (0.27-13.03)<br>aHR 1.96 (0.31, 12.27)<br>Adjusted for: recent incarceration (past 6 months), MMT coverage, crack injecting (past 6 months)
Dumchev et al (unpublished)	**Ukraine cohort,** clients were recruited between March 2013 and December 2013 in 11 Ukrainian cities using respondent-driven sampling method. Participants of the prospective cohort had to meet the following criteria: (1) being HIV negative at the time of screening, confirmed by HIV rapid testing; (2) age of 16 or older by self-report; (3) have visible signs of recent injection, verified by the study nurse; (4) ability to provide informed consent; (5) willingness to participate in the study for 18 months and provide contact information; (6) intention to live in the city of recruitment for 18 months.	Current homelessness	HR 0.00 (0.00-0.00)<br>aHR N/A<br>Adjusted for: Ever prison, age (continuous), IDU duration (continuous).
Hayashi et al (unpublished)[27]	**VIDUS**, The Vancouver Injection Drug Users Study (VIDUS) is an open prospective community-recruited cohort of PWID in Vancouver, Canada. Beginning in May 1996, active PWID (i.e. those who reported injecting drugs in the previous month) were recruited in the Greater Vancouver region on an ongoing basis throughout the study period. Recruitment strategies employ extensive street-based outreach and ''snowball'' sampling approaches. Given VIDUS is an open cohort, new participants were continuously enrolled in the cohort over the study period to replace those who died or were lost to follow-up. All participants were recruited through street outreach, word of mouth, and self-referral, and provided written informed consent prior to entering the study. Individuals were eligible if they had injected illicit drugs at least once in the previous month, were at least 18 years old, and resided in the Vancouver region.	Homelessness in the past 6 months	HR 0.78 (0.54-1.14)<br>aHR 0.73 (0.50-1.06)<br>Adjusted for: recent incarceration (past 6 months), MMT coverage, crack injecting (past 6 months)

Judd et al (unpublished)[46]	**London Cohort,** Recruitment was done in the community settings. In 2001, we recruited from community settings mainly in London, but also in Brighton, 428 injecting drug users who were aged below 30 years or had been injecting for six years or fewer. All had injected in the previous four weeks and could provide addresses for follow up.	Unstable housing in the past 12 months	HR 0.94 (0.23-3.76)
Kral et al. (2001)[65]	**Urban Health Study,** Respondents were not recruited from institutional locations such as drug-treatment programmes, homeless shelters, correctional facilities, clinics, or hospitals. Active IDUs were recruited for the Urban Health Study in three inner-city communities in San Francisco from 1986, and in a fourth from 1996. 6-monthly surveys included 170–250 IDUs in each community. We assessed data from 23 surveys done from 1986 to 1998. Respondents were recruited in natural settings with targeted sampling methods. Communities selected had high concentrations of IDUs according to drug-treatment admission data, police arrest data, direct observation, and earlier ethnographic studies. New respondents were screened for visible signs of recent subcutaneous or intravenous drug use (so-called tracks, or recently punctured veins). Respondents were permitted to participate in subsequent surveys irrespective of whether they had continued to inject drugs. Repeat respondents were identified by checking information against that held in a database on a lap-top computer. Every 6 months, a new sample was recruited; previous participants were not helped to return to the study. This method allowed us to assess new cases of HIV-1 infection from repeat visits of participants. The eligibility criterion was recent intravenous drug use (past 30 days). Respondents were not recruited from institutional locations such as drug-treatment programmes, homeless shelters, correctional facilities, clinics, or hospitals.	Current homelessness	OR 1.24 (0.71-2.17)
Kurth et al (unpublished)[66]	**TLC-IDU,** Study participants were recruited at needle and syringe programs through respondent-driven sampling from ten sites that were implementing partners for Kenya's NSP program working with PWID in Nairobi or Coast regions. Out of the ten study sites, four are in Nairobi and six in Coastal Mombasa. The four study sites in Nairobi, being more urban, are in close proximity, while most of the six study sites in Coastal Mombasa are spread apart from each other. Participants were at least 18 years old, lived in Nairobi or Coast regions, injected non-prescribed drugs at some point in their lifetime, and used non-prescribed drugs by any route of administration in the past year. Potential participants were not enrolled if they were under the influence of substances and thus unable to consent, reported being forced to participate, or if the interviewer was not confident that the potential participant was a PWID based on responses to questions about injection drug use particulars as well as visual observation of the skin.	Current homelessness	IRR 3.45 (1.48-7.62)
Leclerc et al (unpublished)[49]	**SurvUDI,** Participants are recruited in urban areas, including Montréal and neighbouring South Shore, Québec City, the Hull-Ottawa region, and 5 semi-urban areas of the province of Québec. Overall, since 2004, 94.6% of participants were recruited in harm reduction programs. Others were recruited in drop-in centres, detention centres, detoxification clinics, and rehabilitation programmes. Eligibility criteria include being aged 14 years or older, injecting at least once within the past 6 months, speaking French or English and being able to provide informed consent.	Unstable housing in the past 6 months	HR 1.26 (0.83-1.93)<br>aHR 0.98 (0.62-1.53)<br>Adjusted for: Living in jail past 6 months, OST exposure, using syringes used by someone else, Cocaine most often injected drug, Injecting drugs every day, Age >= 25 years, Male gender, Prostitution, Urban sites.
Mehta et al (unpublished)[29]	**ALIVE,** Participants were recruited into the study from various agencies that served intravenous drug users. These agencies include Maryland Division of Parole and Probation, the Baltimore City Health Department's STD clinics, hospital emergency rooms, and homeless shelters in Baltimore. To extend recruitment beyond these agencies, outreach efforts were developed in collaboration with the Street Outreach AIDS Prevention (SOAP) Unit of the Health Education Resource Organization (HERO), a community AIDS education group in Maryland. The SOAP unit include ten recovered and recovering addicts who provide AIDS education to the inner-city community through contacts on the street. These workers distributed brochures and answered questions about the ALIVE study. Study staff also distributed brochures at local public housing projects and other public places known to be frequented by IVDUs. In summary, recruitment sources include: Word-of-mouth, drug treatment programs, HERO Street Outreach, Parole and Probation, STD Clinics, HIV clinics, and emergency rooms. Between 1998-1999, all participants acknowledged non- medical injection-drug use within the preceding 11 years, were >18 years of age, and were free of AIDS at entry into the study. Additional persons were recruited into this cohort in 1994–1995 (n 5 391), 1998 (n 5 244), and 2005–2008 (n 5 875). Some recruitment criteria changed over time. In the fourth period, persons were no longer required to be AIDS-free at entry. To replenish with active injectors, in 1994–1995, persons had to have injected in the preceding 3 years, and in 1998 and 2005–2008, in the pre- ceding year."	Homelessness in the past 6 months	IRR 1.58 (1.15-2.17)<br>aIRR 1.16 (0.84-1.60)<br>Adjusted for: Cocaine, Jail, OST/MAT treatment
Mehta et al (unpublished)[67]	**Indian ICC Study,** at each site, we partnered with nongovernmental organizations that provide services to PWIDs and conducted preliminary ethnographic work. We used respondent-driven sampling to recruit PWIDs, with the goal of recruiting 1000 participants from each site [14 – 16]. We initiated recruitment at each site with two or three 'seeds' – individuals identified in the ethnographic phase as well connected in the PWID communities. Individuals were eligible to participate if they were 18 years or older, reported	Current homelessness	IRR 1.56 (0.90-2.70)<br>aIRR 1.52 (0.88-2.63)<br>Adjusted for: injected stimulants or crack/cocaine in prior 6 months

	injecting drug use in the prior 2 years, provided verbal informed consent, and presented a valid recruitment coupon (except for the seeds). We excluded data from 'seed' participants from the analyses.		[yes/no], participated in OST program in prior 6 months [yes/no], incarcerated in prior 6 months [yes/no].
Niccolai et al. (2011)[68]	**SATHCAP,** Participants were recruited into the study using respondent-driven sampling, a chain referral sampling method that uses dual incentives and structured coupon disbursement procedures for peer referrals. Eligibility for inclusion in the present analysis included reporting a history of ever injecting drugs. No cohort exclusion criteria but exclusion at BED EIA analysis stage, Correlates of incident infections stage and Spatial patterns stage.	Homelessness in the past 12 months	RR 0.70 (0.33-1.52)
Samo et al. (2013)[69]	We conducted our study at three drop-in centres that provide basic harm reduction and social services to PWID exclusive of opiate substitution therapy. Excluded eight persons who were unable to understand the study objectives due to disabilities that precluded provision of informed consent.	Current homelessness	IRR 1.70 (1.20-2.50)<br>aIRR 1.70 (1.10-2.50)<br>Adjusted for: sharing of syringes (yes vs no), non-Muslim religion (non-Muslims vs Muslims), daily frequency of injecting drugs (#/day), source of registration (others vs outreach), physical disability (yes vs no), monthly income (<5000 Pakistani rupees vs >= 5000 Pakistani rupees) and sources of syringes/needles (other vs DIC & MSU).
Strathdee et al (unpublished)[70]	**El Cuete III,** Respondent-driven sampling (RDS) was used to recruit participants. Briefly, a diverse group of "seeds" (heterogeneous by age, gender, and neighbourhood) was selected and given uniquely coded coupons to refer their peers to the study. Waves of recruitment continued as subjects returning with coupons were given coupons to recruit members of their social networks. Recruitment and interviews were conducted by indigenous outreach workers through the use of a modified recreational vehicle and a storefront office. Eligibility criteria included being ≥18 years of age; having injected illicit drugs within the past month, as confirmed by inspection of injection stigmata ("track-marks"); ability to speak Spanish or English; being able to provide informed consent; and having no plans to permanently move out of the city in the next 18 months.	Unstable housing in the past 6 months	HR 1.50 (0.55-4.07)
Strathdee et al (unpublished)[71]	**El Cuete IV,** we sought to obtain representative samples of PWID in each site. Although we initially considered recruiting new cohorts of PWID using respondent driven sampling (RDS), due to issues of cost and the limited effectiveness of RDS at recruiting female PWID, we instead used targeted sampling consisting of street-based out-reach in diverse geographic areas For example, El Cuete IV outreach teams established temporary mobile recruitment sites (e.g., vans and tents) in ten distinct colonias (neighbourhoods) characterized by different physical risk environments and where PWID were known to spend time. Once situated in these neighbourhoods, outreach workers attempted to engage individuals in conversation, sometimes by offering HIV prevention materials or information (e.g., condoms, educational pamphlets). In both studies, eligibility criteria included the following characteristics: being at least 18 years of age, having evidence of injecting illicit drugs within the past month (i.e., confirmed by observation of track marks or other physical evidence of injecting), 3) being able to converse in English or Spanish, 4) currently residing in the study city with no plans to move away within 24 months from enrolment date, and 5) not currently participating in any intervention studies (although none to our knowledge were being conducted). Individuals with severe cognitive deficiencies or who were unwilling to provide informed consent were excluded, and PWID who met eligibility criteria but were too intoxicated to provide informed consent were rescheduled for rescreening at a later date.	Unstable housing in the past 6 months	HR 2.10 (1.13-3.90)
Sypsa et al. (2017)[58]	**ARISTOTLE,** Participants were recruited through respondent-driven sampling. A dual incentive system was used in which participants received incentives for participating in the programme (primary incentives) as well as for recruiting others (secondary incentives). Seeds were recruited by staff of the Greek Organization Against Drugs (OKANA) who had experience in working with the target population. The programme itself was implemented in a building of the OKANA, located in the centre of Athens. The staff included a physician, inter-viewers with prior experience with the target population and a psychologist and two social workers. A flow manager was responsible for maintaining the flow of participants and ensuring that participants completed each of the steps of the process on a first-come, first-served basis. Five RDS rounds were implemented. The selection of seeds was based on whether the candidates were well connected to other members of the target population, liked by their peers and with motivation to the programme.	Current homelessness	HR 1.75 (1.30-2.36)<br>aHR 1.96 (0.98-3.85)<br>Adjusted for: age (<=35 vs >35), sex (male vs female), country of origin (Greece vs middle east vs others), History of any imprisonment (No vs Yes), Size of participant's network PWID (1-10 vs 11-30 vs >30),

	The aim was to enrol seeds diverse in terms of country of origin, gender and HIV status to ensure reaching equilibrium. Respondents were eligible to participate if they presented a valid RDS coupon, had injected drugs without a prescription in the past 12 months, were 18 years of age and over, and resided in the Athens metropolitan area. A subset of PWID participated in multiple RDS rounds, allowing for assessment of HIV seroconversion.		currently on OST program (Yes vs No), Injecting drug use behaviour: main substance of use (Heroin/Thai vs Cocaine/speedball), injecting drug use in past 1 month(No vs Yes), frequency of drug use (less than once weekly vs at least once weekly vs at least once daily), sharing syringes(never or rarely vs about half the time or more), use of drugs divided with a syringe that someone else had already used for injection (never or rarely vs about half the time or more).
Todd et al (unpublished)[60]	**Kabul Harm Reduction Program,** Participants were recruited consecutively from areas of known drug user congregation and through harm reduction programs on scheduled days loosely apportioned by number of IDUs present at those locations, in a variant of time-location sampled. Recruitment sites were initially determined through formative work and through harm reduction programs, with locations adjusted monthly based on information from the participants, the harm reduction field workers, and pharmacists of new areas of congregation. Sites were distributed throughout Kabul city, with most sites in the western section of the city, consistent with reported and confirmed IDU presence throughout the study period. Eligibility was limited to IDUs who were aged ≥18 years; reported injecting drugs within the prior 30 days; residing in Kabul; Dari or Pashto speakers; and able to provide informed consent.	Homelessness in the past 6 months	HR 0.45 (0.05-3.91)
Van Santen et al (unpublished)[62]	**Amsterdam Cohort Study,** Participants were recruited mainly through low-threshold methadone programs (including a weekly sexually transmitted disease clinic for drug-using sex workers) and by word of mouth.	Homelessness in the past 6 months	HR 2.02 (1.01-4.02)<br>aHR 2.02 (1.01-4.01)<br>Adjusted for: methadone dosing [no methadone vs <60mg vs >=60mg; time updated].

Appendix table 7: Risk of Bias Assessment for the effect of recent homelessness or unstable housing on HCV acquisition risk.

Author and Year of publication	Representativeness of exposed cohort	Selection of non-exposed cohort	Ascertainment of exposure	Demonstration that outcome of interest was not present at the start of study	Comparability of cohorts on the basis of the design or analysis	Assessment of outcome	Was follow-up long enough for outcome to occur	Adequacy of follow-up of cohort	Total (/9)
Artenie et al (2019)[40]		*	*	*	**	*	*	*	**8**
Craine et al. (2009)[41]	*	*	*	*	*	*	*		**7**
Debeck et al (unpublished)[24]		*	*	*	**	*	*	*	**8**
Dumchev et al (unpublished)		*	*	*		*	*	*	**6**
Hayashi et al (unpublished)[27]	*	*	*	*	**	*	*	*	**9**
Hagan et al. (2001)[42]		*	*	*		*	*		**5**
Hagan et al. (2010)[43]		*	*	*		*	*		**5**
Hope et al. (2018)[44]		*	*			*			**3**
Hope et al (unpublished)[45]	*	*	*		**	*			**6**
Judd et al (unpublished)[46]		*	*	*		*	*		**5**
Kaberg et al (unpublished)[47]		*	*	*		*	*		**5**
La Rosa et al (unpublished)[48]		*	*	*	**	*	*		**7**
Leclerc et al (unpublished)[49]		*	*	*	**	*	*		**7**
Lucidarme et al. (2004)[50]		*	*	*		*	*		**5**
Maher et al (unpublished)[51]	*	*	*	*		*	*		**6**
Maher et al (unpublished)[52]	*	*	*	*		*	*	*	**7**
Mehta et al (unpublished)[29]	*	*	*	*	**	*	*	*	**9**
Morris (unpublished)[53]		*	*	*		*	*		**5**

Palmateer et al. (2014) [54]	*	*	*		*	*			**5**
Sacks-Davis et al (unpublished)[55]		*	*	*	*	*	*		**6**
Schulkind et al. (2019)[56]		*	*	*		*	*	*	**6**
Spittal et al. (2012)[57]		*	*	*		*	*		**5**
Sypsa et al (unpublished)[58]		*	*	*		*	*		**5**
Thorpe et al. (2002)[59]		*	*	*	*	*	*		**6**
Todd et al (unpublished)[60]	*	*	*	*		*	*		**6**
Vallejo et al. (2015)[61]		*	*	*		*	*		**5**
Van Santen et al (unpublished)[62]		*	*	*	*	*	*		**6**
Wijnand et al (unpublished)[63]	*	*	*	*	**	*	*		**8**

Appendix table 8: Risk of Bias Assessment for the effect of recent homelessness or unstable housing on HIV acquisition risk.

Author and Year of publication	Representativeness of exposed cohort	Selection of non-exposed cohort	Ascertainment of exposure	Demonstration that outcome of interest was not present at the start of study	Comparability of cohorts on the basis of the design or analysis	Assessment of outcome	Was follow-up long enough for outcome to occur	Adequacy of follow-up of cohort	Total (/9)
Bruneau et al. (2011)[64]	*	*	*	*	*	*	*		**7**
Debeck et al (unpublished)[24]		*	*	*	**	*	*	*	**8**
Dumchev et al (unpublished)		*	*	*		*	*	*	**6**
Hayashi et al (unpublished)[27]		*	*	*	**	*	*	*	**8**
Judd et al (unpublished)[46]		*	*	*		*	*		**5**
Kral et al. (2001)[65]		*	*	*		*			**4**
Kurth et al (unpublished)[66]		*	*	*		*	*		**5**
Leclerc et al (unpublished)[49]		*	*	*	**	*	*		**7**
Mehta et al (unpublished)[29]	*	*	*	*	**	*	*		**8**
Mehta et al (unpublished)[67]	*	*	*		**	*			**6**
Niccolai et al. (2011)[68]	*	*	*			*			**4**
Samo et al. (2013)[69]		*	*	*		*	*		**5**
Strathdee et al (unpublished)[70]	*	*	*	*		*	*	*	**7**
Strathdee et al (unpublished)[71]		*	*	*		*	*		**5**
Sypsa et al. (2017)[58]	*	*	*	*	**	*	*		**8**
Todd et al (unpublished)[60]	*	*	*	*		*	*		**6**
Van Santen et al (unpublished)[62]		*	*	*	*	*	*		**6**

Acknowledgements: CA received funding from Commonwealth Scholarship Commission for his master's degree. PV, HF and JS acknowledge support from U.S. National Institute for Drug Abuse (NIDA grant number R01 DA033679, R01 DA037773, R21 DA046809 and R01 DA047952) and NIAID/NIDA (R01AI147490). PV, ZW, HF, JS and MH are supported by the National Institute for Health Research Health Protection Research Units (NIHR HPRUs) in Evaluation of Interventions and Behavioural Science at the University of Bristol in partnership with Public Health England (PHE). MH, PV, and HF also acknowledges support from the NIHR funded EPIToPe project. AAA is supported through postdoctoral fellowships through the Canadian Institute of Health Research, Fonds de recherche du Québec – Santé and Canadian Network on Hepatitis C. KH acknowledges support from NIDA (U01DA038886). AK acknowledges support from NIDA (R01DA032080). KP acknowledges support from NIDA (R01 DA016017). LM is supported by Australian National Health and Medical Research Council (NHMRC) Research Fellowship GNT1154839. SM acknowledges support from NIDA (R01DA041034 and R01DA036297). JJO acknowledges support from Australian National Health and Medical Research Council (APP1104781). CST acknowledges funding for the Afghanistan cohort from the Doris Duke Charitable Foundation. MHellard receives research funding and a fellowship from the Australian National Health and Medical Research Council. PL and MA acknowledge funding from the Public Health Agency of Canada and the Ministère de la santé et des services sociaux du Québec. MM acknowledges funding from NIDA (K01DA037802).

The views expressed in this publication are those of the authors and not necessarily those of the National Health Service, the NIHR, the Department of Health, Public Health England, U.S. National Institute on Drug Abuse or the National Institutes of Health (NIH).

References

1. Aitken C K, Lewis J, Tracy S L, et al. High incidence of hepatitis C virus reinfection in a cohort of injecting drug users. *Hepatology. 2008*;48(6):1746-52.
2. Allen E J, Palmateer N E, Hutchinson S J, Cameron S, Goldberg D J, Taylor A. Association between harm reduction intervention uptake and recent hepatitis C infection among people who inject drugs attending sites that provide sterile injecting equipment in Scotland. *International Journal of Drug Policy. 2012*;23(5):346-52.
3. Bach P, Walton G, Hayashi K, et al. Benzodiazepine Use and Hepatitis C Seroconversion in a Cohort of Persons Who Inject Drugs. *American Journal of Public Health. 2016*;106(6):1067-72.
4. Boileau C, Bruneau J, Al-Nachawati H, Lamothe F, Vincelette J. A prognostic model for HIV seroconversion among injection drug users as a tool for stratification in clinical trials. *Journal of Acquired Immune Deficiency Syndromes. 2005*;39(4):489-95.
5. Bruneau J, Roy E, Arruda N, Zang G, Jutras-Aswad D. The rising prevalence of prescription opioid injection and its association with hepatitis C incidence among street-drug users. *Addiction. 2012;*107(7):1318-27.
6. Bruneau J, Daniel M, Zang G, Lamothe F, Vincelette J. Decrease of HIV incidence among injection drug users (IDU) in Montreal: Evidence from the saint-Luc cohort. *Canadian Journal of Infectious Diseases and Medical Microbiology. 2010*; SB):21B.
7. Bruneau J, Roy E, Arruda N, Zang G, Jutras-Aswad D. The rising prevalence of prescription opioid injection and its association with hepatitis C incidence among street-drug users. *Addiction. 2012*;107(7):1318-27.
8. Corneil T A, Kuyper L M, Shoveller J, et al. Unstable housing associated risk behaviour, and increased risk for HIV infection among injection drug users. *Health AND Place. 2006*;12(1):79-85.
9. Craib K J, Spittal P M, Wood E, et al. Risk factors for elevated HIV incidence among Aboriginal injection drug users in Vancouver. *CMAJ. 2003*;168(1):19-24.
10. Cullen K J, Hope V D, Croxford S, Shute J, Ncube F, Parry JV. Factors associated with recently acquired hepatitis C virus infection in people who inject drugs in England, Wales and Northern Ireland: new findings from an unlinked anonymous monitoring survey. *Epidemiology AND Infection. 2015*;143(7):1398-407.
11. Grebely J, Lima V D, Marshall B D, et al. Declining incidence of hepatitis C virus infection among people who inject drugs in a Canadian setting, 1996-2012. *PLoS ONE. 2014*;9(6): e97726.
12. Hahn J A, Page-Shafer K, Lum P J, et al. Hepatitis C virus seroconversion among young injection drug users: relationships and risks. *Journal of Infectious Diseases. 2002*;186(11):1558-64.
13. Ickowicz S, Hayashi K, Dong H, et al. Benzodiazepine use as an independent risk factor for HIV infection in a Canadian setting. *Drug AND Alcohol Dependence. 2015*; 155:190-4.
14. Jacka B, Roy E, Hoj S, et al. Sexual behaviour as a risk factor for hepatitis C virus infection among people who inject drugs in Montreal, Canada. *J Viral Hepat 2019;* 26: 1413 – 1422.
15. Kaberg M, Naver G, Hammarberg A, Weiland O. Incidence, and spontaneous clearance of hepatitis C virus (HCV) in people who inject drugs at the Stockholm Needle Exchange-Importance for HCV elimination. *Journal of Viral Hepatitis. 2018*;25(12):1452-61.
16. Kim C, Kerr T, Li K, et al. Unstable housing and hepatitis C incidence among injection drug users in a Canadian setting. *BMC Public Health. 2009*; 9:270.
17. Maas B, Fairbairn N, Kerr T, Li K, Montaner JS, Wood E. Neighbourhood and HIV infection among IDU: place of residence independently predicts HIV infection among a cohort of injection drug users. *Health AND Place. 2007*;13(2):432-9.
18. Maher L, Jalaludin B, Chant K G, et al. Incidence and risk factors for hepatitis C seroconversion in injecting drug users in Australia. *Addiction. 2006*;101(10):1499-508.
19. Miller C L, Kerr T, Frankish J C, et al. Binge drug use independently predicts HIV seroconversion among injection drug users: implications for public health strategies. *Substance Use AND Misuse. 2006*;41(2):199-210.
20. Miller C L, Strathdee S A, Spittal P M, et al. Elevated rates of HIV infection among young Aboriginal injection drug users in a Canadian setting. *Harm Reduction Journal. 2006*; 3:9.

21. Miller C L, Tyndall M, Spittal P, Li K, LaLiberte N, Schechter M T. HIV incidence and associated risk factors among young injection drug users. *AIDS. 2002*;16(3):491-3.
22. Morris M D, Shiboski S, Bruneau J, et al. Geographic differences in temporal incidence trends of hepatitis C virus infection among people who inject drugs: The InC3 collaboration. *Clinical Infectious Diseases. 2017*;64(7):860-9.
23. Nelson K E, Galai N, Safaeian M, Strathdee S A, Celentano D D, Vlahov D. Temporal trends in the incidence of human immunodeficiency virus infection and risk behaviour among injection drug users in Baltimore, Maryland, 1988-1998. *American Journal of Epidemiology. 2002*;156(7):641-53
24. Puri N, DeBeck K, Feng C, Kerr T, Rieb L, Wood E. Gender influences on hepatitis C incidence among street youth in a Canadian setting. *Journal of Adolescent Health. 2014*;55(6):830-4.
25. Puzhko S, Roy E, Jutras-Aswad D, et al. High hepatitis C incidence in relation to prescription opioid injection and poly-drug use: Assessing barriers to hepatitis C prevention. *International Journal of Drug Policy. 2017*; 47:61-8.
26. Sacks-Davis R, Daniel M, Roy E, et al. The role of living context in prescription opioid injection and the associated risk of hepatitis C infection. *Addiction. 2016*;111(11):1985-96.
27. Scheim A I, Nosova E, Knight R, Hayashi K, Kerr T. HIV Incidence Among Men Who Have Sex with Men and Inject Drugs in a Canadian Setting. *Aids Behav. 2018*;22(12):3957-61.
28. Shannon K, Kerr T, Marshall B, et al. Survival sex work involvement as a primary risk factor for hepatitis C virus acquisition in drug-using youths in a Canadian setting. *Arch Pediatr Adolesc Med. 2010*;164(1):61-5.
29. Smith D K, Pan Y, Rose C E, et al. A Brief Screening Tool to Assess the Risk of Contracting HIV Infection Among Active Injection Drug Users. *Journal of Addiction Medicine. 2015*;9(3):226-32.
30. Spittal P M, Craib KJ, Wood E, et al. Risk factors for elevated HIV incidence rates among female injection drug users in Vancouver. *CMAJ. 2002;*166(7):894-9.
31. Strathdee S A, Galai N, Safaiean M, et al. Sex differences in risk factors for HIV seroconversion among injection drug users: a 10-year perspective. *Arch Intern Med. 2001*;161(10):1281-8.
32. Tyndall M W, Currie S, Spittal P, et al. Intensive injection cocaine use as the primary risk factor in the Vancouver HIV-1 epidemic. *AIDS. 2003;*17(6):887-93.
33. Van den Berg C H, Smit C, Bakker M, et al. Major decline of hepatitis C virus incidence rate over two decades in a cohort of drug users. *Eur J Epidemiol. 2007*;22(3):183-93.
34. Van Den Berg C, Smit C, Van Brussel G, Coutinho R, Prins M, Amsterdam C. Full participation in harm reduction programmes is associated with decreased risk for human immunodeficiency virus and hepatitis C virus: evidence from the Amsterdam Cohort Studies among drug users. *Addiction. 2007*;102(9):1454-62.
35. Tsui J I, Evans J L, Lum P J, Hahn JA, Page K. Association of opioid agonist therapy with lower incidence of hepatitis C virus infection in young adult injection drug users. *JAMA Internal Medicine. 2014*;174(12):1974-81.
36. White B, Madden A, Prins M, et al. Assessing the feasibility of hepatitis C virus vaccine trials: results from the Hepatitis C Incidence and Transmission Study-community (HITS-c) vaccine preparedness study. *Vaccine. 2014*;32(42):5460-7.
37. Wood E, Kerr T, Marshall B D, et al. Longitudinal community plasma HIV-1 RNA concentrations and incidence of HIV-1 among injecting drug users: prospective cohort study. *BMJ. 2009*; 338: b1649.
38. Wood E, Lloyd-Smith E, Li K, et al. Frequent needle exchange use and HIV incidence in Vancouver, Canada. *American Journal of Medicine. 2007*;120(2):172-9.
39. Young S V, Wood E, Dong H, Kerr T, Hayashi K. Daily alcohol use as an independent risk factor for HIV seroconversion among people who inject drugs. *Addiction. 2016*;111(8):1360-5.
40. Artenie A A, Minoyan N, Jacka B, et al. Opioid agonist treatment dosage and patient-perceived dosage adequacy, and risk of hepatitis C infection among people who inject drugs. *CMAJ. 2019*;191(17): E462-E8.
41. Craine N, Hickman M, Parry JV, et al. Incidence of hepatitis C in drug injectors: the role of homelessness, opiate substitution treatment, equipment sharing, and community size. *Epidemiology AND Infection. 2009*;137(9):1255-65.

42. Hagan H, Thiede H, Weiss N S, Hopkins S G, Duchin J S, Alexander E R. Sharing of drug preparation equipment as a risk factor for hepatitis C. *American Journal of Public Health. 2001*;91(1):42-6.
43. Hagan H, Pouget E R, Williams I T, et al. Attribution of hepatitis C virus seroconversion risk in young injection drug users in 5 US cities. *Journal of Infectious Diseases. 2010;*201(3):378-85.
44. Hope V D, Harris R J, Vickerman P, et al. A comparison of two biological markers of recent hepatitis C virus (HCV) infection: implications for the monitoring of interventions and strategies to reduce HCV transmission among people who inject drugs. *Euro Surveill 2018*; 23(47).
45. Turner K M, Hutchinson S, Vickerman P, et al. The impact of needle and syringe provision and opiate substitution therapy on the incidence of hepatitis C virus in injecting drug users: pooling of UK evidence. *Addiction. 2011*;106(11):1978-88.
46. Judd A, Hickman M, Jones S, McDonald T, Parry JV, Stimson GV, et al. Incidence of hepatitis C virus and HIV among new injecting drug users in London: Prospective cohort study. *BMJ: British Medical Journal. 2005*;330(7481):24-5.
47. Kaberg M, Naver G, Hammarberg A, Weiland O. Incidence and spontaneous clearance of hepatitis C virus (HCV) in people who inject drugs at the Stockholm Needle Exchange-Importance for HCV elimination. *Journal of Viral Hepatitis. 2018*;25(12):1452-61.
48. La Rosa J V, Ryan P, Alvaro-Meca A, et al. HCV seroconversion in a cohort of people who use drugs followed in a mobile harm reduction unit in Madrid: Breaking barriers for HCV elimination. *PLoS ONE. 2018*;13 (10) (no pagination) (e0204795).
49. Blouin K, Leclerc P, Morissette C, et al. Sex Work as an Emerging Risk Factor for Human Immunodeficiency Virus Seroconversion Among People Who Inject Drugs in the SurvUDI Network. *Sexually Transmitted Diseases. 2016*;43(10):648-55.
50. Lucidarme D, Bruandet A, Ilef D, et al. Incidence and risk factors of HCV and HIV infections in a cohort of intravenous drug users in the North and East of France. *Epidemiology AND Infection. 2004*;132(4):699-708.
51. Maher L, Jalaludin B, Chant KG, et al. Incidence and risk factors for hepatitis C seroconversion in injecting drug users in Australia. *Addiction. 2006*;101(10):1499-508.
52. White B, Madden A, Prins M, et al. Assessing the feasibility of hepatitis C virus vaccine trials: results from the Hepatitis C Incidence and Transmission Study-community (HITS-c) vaccine preparedness study. *Vaccine. 2014*;32(42):5460-7.
53. Page K, Hahn J A, Evans J, et al. Acute hepatitis C virus infection in young adult injection drug users: a prospective study of incident infection, resolution, and reinfection. *Journal of Infectious Diseases. 2009*;200(8):1216-26.
54. Palmateer N E, Taylor A, Goldberg D J, et al. Rapid decline in HCV incidence among people who inject drugs associated with national scale-up in coverage of a combination of harm reduction interventions. *PLoS ONE. 2014*;9(8): e104515.
55. Sacks-Davis R, Aitken CK, Higgs P, Spelman T, Pedrana AE, Bowden S, et al. High rates of hepatitis C virus reinfection and spontaneous clearance of reinfection in people who inject drugs: a prospective cohort study. PLoS ONE. 2013;8(11): e80216.
56. Schulkind J, Stephens B, Ahmad F, et al. High response and re-infection rates among people who inject drugs treated for hepatitis C in a community needle and syringe program. *J Viral Hepat 2019:*26: 519-528.
57. Spittal P M, Pearce M E, Chavoshi N, et al. The Cedar Project: high incidence of HCV infections in a longitudinal study of young Aboriginal people who use drugs in two Canadian cities. *BMC Public Health. 2012*; 12:632.
58. Sypsa V, Psichogiou M, Paraskevis D, et al. Rapid Decline in HIV Incidence Among Persons Who Inject Drugs During a Fast-Track Combination Prevention Program After an HIV Outbreak in Athens. *J Infect Dis 2017*; 215(10): 1496-505.
59. Thorpe L E, Ouellet L J, Hershow R, et al. Risk of hepatitis C virus infection among young adult injection drug users who share injection equipment. *American Journal of Epidemiology. 2002*;155(7):645-53.
60. Todd C S, Nasir A, Stanekzai M R, et al. Hepatitis C and HIV incidence and harm reduction program use in a conflict setting: an observational cohort of injecting drug users in Kabul, Afghanistan. *Harm Reduction Journal. 2015*; 12:22.

61. Vallejo F, Barrio G, Brugal M T, et al. High hepatitis C virus prevalence and incidence in a community cohort of young heroin injectors in a context of extensive harm reduction programmes. *J Epidemiol Community Health. 2015*;69(6):599-603.
62. Van Den Berg C, Smit C, Van Brussel G, Coutinho R, Prins M, Amsterdam C. Full participation in harm reduction programmes is associated with decreased risk for human immunodeficiency virus and hepatitis C virus: evidence from the Amsterdam Cohort Studies among drug users. *Addiction. 2007*;102(9):1454-62.
63. Horyniak D, Higgs P, Jenkinson R, Degenhardt L, Stoove M, Kerr T, Hickman M, Aitken C, Dietze P. Establishing the Melbourne Injecting Drug User Cohort Study (MIX): rationale, methods, and baseline and twelve-month follow-up results. *Harm Reduct J. 2013*; 10:11.
64. Bruneau J, Daniel M, Abrahamowicz M, Zang G, Lamothe F, Vincelette J. Trends in human immunodeficiency virus incidence and risk behaviour among injection drug users in Montreal, Canada: a 16-year longitudinal study. *American Journal of Epidemiology. 2011*;173(9):1049-58.
65. Kral A H, Bluthenthal R N, Lorvick J, Gee L, Bacchetti P, Edlin B R. Sexual transmission of HIV-1 among injection drug users in San Francisco, USA: risk-factor analysis. *Lancet. 2001*;357(9266):1397-401.
66. Kurth A E, Cleland C M, Des Jarlais D C, et al. HIV prevalence, estimated incidence, and risk behaviours among people who inject drugs in Kenya. *J Acquir Immune Defic Syndr. 2015*;70(4);420-7.
67. Lucas G M, Solomon S S, Srikrishnan A K, et al. High HIV burden among people who inject drugs in 15 Indian cities. *AIDS 2015*; 29:619-628.
68. Niccolai L M, Verevochkin S V, Toussova O V, et al. Estimates of HIV incidence among drug users in St. Petersburg, Russia: continued growth of a rapidly expanding epidemic. *Eur J Public Health. 2011*;21(5):613-9.
69. Samo R N, Altaf A, Agha A, et al. High HIV incidence among persons who inject drugs in Pakistan: greater risk with needle sharing and injecting frequently among the homeless. *PLoS ONE. 2013*;8(12): e81715.
70. Robertson A M, Garfein R S, Wagner K D, et al. Evaluating the impact of Mexico’s drug policy reforms on people who inject drugs in Tijuana, B.C., Mexico, and San Diego, CA United State: a binational mixed methods research agenda. *Harm Reduction Journal 2014*; 11:4.
71. Strathdee S A, Lozada R, Pollini R A, et al. Individual, social, and environmental influences associated with HIV infection among injection drug users in Tijuana, Mexico. *J Acquir Immune Defic Syndr. 2008*; 47(3): 369-376.
